# Comprehensive temporal analysis of right ventricular function and pulmonary haemodynamics in mechanically ventilated COVID-19 ARDS patients

**DOI:** 10.1186/s13613-024-01241-1

**Published:** 2024-02-12

**Authors:** Vasiliki Tsolaki, George E. Zakynthinos, Nikitas Karavidas, Vasileios Vazgiourakis, John Papanikolaou, Kyriaki Parisi, Paris Zygoulis, Demosthenes Makris, Epaminondas Zakynthinos

**Affiliations:** 1 Critical Care Department, Faculty of Medicine, University Hospital of Larissa, University of Thessaly, Mezourlo, 41110 Larissa, Greece; 2 Third Cardiology Clinic, University of Athens, Sotiria Hospital, Athens, Greece; 3 Department of Cardiology, General Hospital of Trikala, Karditsis 56, 42131 Trikala, Thessaly Greece

**Keywords:** COVID-19, Cardiac function, RV dysfunction, Pulmonary vascular resistance, PEEP, ARDS, Hemodynamics, Strain

## Abstract

**Background:**

Cardiac injury is frequently reported in COVID-19 patients, the right ventricle (RV) is mostly affected. We systematically evaluated the cardiac function and longitudinal changes in severe COVID-19 acute respiratory distress syndrome (ARDS) admitted to the intensive care unit (ICU) and assessed the impact on survival.

**Methods:**

We prospectively performed comprehensive echocardiographic analysis on mechanically ventilated COVID-19 ARDS patients, using 2D/3D echocardiography. We defined left ventricular (LV) systolic dysfunction as ejection fraction (EF) < 40%, or longitudinal strain (LS) > − 18% and right ventricular (RV) dysfunction if two indices among fractional area change (FAC) < 35%, tricuspid annulus systolic plane excursion (TAPSE) < 1.6 cm, RV EF < 44%, RV–LS > − 20% were present. RV afterload was assessed from pulmonary artery systolic pressure (PASP), PASP/Velocity Time Integral in the right ventricular outflow tract (VTI_*RVOT*_) and pulmonary acceleration time (PAcT). TAPSE/PASP assessed the right ventriculoarterial coupling (VAC_*R*_).

**Results:**

Among 176 patients included, RV dysfunction was common (69%) (RV–EF 41.1 ± 1.3%; RV–FAC 36.6 ± 0.9%, TAPSE 20.4 ± 0.4mm, RV–LS:− 14.4 ± 0.4%), usually accompanied by RV dilatation (RVEDA/LVEDA 0.82 ± 0.02). RV afterload was increased in most of the patients (PASP 33 ± 1.1 mmHg, PAcT 65.3 ± 1.5 ms, PASP/VTI_*RVOT*_, 2.29 ± 0.1 mmHg/cm). VAC_*R*_ was 0.8 ± 0.06 mm/mmHg. LV–EF < 40% was present in 21/176 (11.9%); mean LV–EF 57.8 ± 1.1%. LV–LS (− 13.3 ± 0.3%) revealed a silent LV impairment in 87.5%. A mild pericardial effusion was present in 70(38%) patients, more frequently in non-survivors (*p* < 0.05). Survivors presented significant improvements in respiratory physiology during the 10th ICU-day (PaO_2_/FiO_2_, 231.2 ± 11.9 vs 120.2 ± 6.7 mmHg; PaCO_2_, 43.1 ± 1.2 vs 53.9 ± 1.5 mmHg; respiratory system compliance—C_*RS*_, 42.6 ± 2.2 vs 27.8 ± 0.9 ml/cmH_2_O, all *p* < 0.0001). Moreover, survivors presented significant decreases in RV afterload (PASP: 36.1 ± 2.4 to 20.1 ± 3 mmHg, *p* < 0.0001, PASP/VTI_*RVOT*_: 2.5 ± 1.4 to 1.1 ± 0.7, *p* < 0.0001 PAcT: 61 ± 2.5 to 84.7 ± 2.4 ms, *p* < 0.0001), associated with RV systolic function improvement (RVEF: 36.5 ± 2.9% to 46.6 ± 2.1%, *p* = 0.001 and RV–LS: − 13.6 ± 0.7% to − 16.7 ± 0.8%, *p* = 0.001). In addition, RV dilation subsided in survivors (RVEDA/LVEDA: 0.8 ± 0.05 to 0.6 ± 0.03, *p* = 0.001). Day-10 C_*RS*_ correlated with RV afterload (PASP/VTI_*RVOT*_, r: 0.535, *p* < 0.0001) and systolic function (RV–LS, 0.345, *p* = 0.001). LV–LS during the 10th ICU-day, while ΔRV–LS and ΔPASP/RVOT_*VTI*_ were associated with survival.

**Conclusions:**

COVID-19 improvements in RV function, RV afterload and RV–PA coupling at day 10 were associated with respiratory function and survival.

**Supplementary Information:**

The online version contains supplementary material available at 10.1186/s13613-024-01241-1.

## Background

Acute cardiac injury is the most common cardiac abnormality in Coronavirus disease 2019 (COVID-19) Acute Respiratory Distress Syndrome (ARDS), mostly defined by cardiac troponin elevation > 99th percentile or basic echocardiographic data [1–5]. The incidence is more common in critically ill patients (around 50%) compared to hospitalized patients in the general wards (20%) [6, 7]. The proposed pathogenetic mechanisms include direct myocardial injury, myocardial oxygen supply–demand imbalance, increased right ventricular (RV) afterload, diffuse endothelialitis and procoagulant activity [8].

The ECHO–COVID study reported that among Intensive Care Units (ICU) patients, mechanically ventilated (MV) or not, 34.5% presented RV and 22% had left ventricular (LV) systolic dysfunction, based on rough visual estimation [9]. A large worldwide survey presenting data through basic echocardiography in COVID-19 patients, indicated that echocardiography may change the management in 33% of the cases [4]. However, more detailed examinations are still rather scarce, especially in MV patients [5, 10, 11]; moreover, longitudinal changes of myocardial damage in MV COVID-19 ARDS patients during the course of ICU stay, have been recently reported in two studies, pointing that different degrees of RV impairment might affect mortality [12, 13]. Considering that the median reported intubation time is 10–14 days [14–17] and that stress cardiomyopathy alleviates by the 7th–10th day after onset [18, 19] we hypothesized that survivors may have improved RV function by that time.

Thus, the aim of the present study is to systematically evaluate the cardiac function and its temporal changes by the 10th ICU day, in intubated COVID-19 ARDS patients using conventional, speckle tracking and three-dimensional (3D) echocardiography and troponin levels. Second, we investigated the possible effects of COVID-19 cardiac function on survival.

## Methods

### Study population

From 4/2020 to 6/2022, we prospectively evaluated the cardiac function in consecutive MV patients, intubated due to respiratory failure from COVID-19 ARDS, from the University Hospital of Larissa, Greece. This study was approved by the University Hospital of Larissa Ethics Board (Cardiac function in mechanically ventilated COVID-19 ARDS patients, 16965/2020), with a waiver for informed consent, as the assessment of cardiac function was part of the routine care of patients admitted in our ICU. The procedures followed are in accordance with the ethical standards of the local institutional review board and the Helsinki Declaration of 1975. Exclusion criteria were: (1) severe stenosis and/or regurgitation of the aortic valve; (2) pre-existing severe heart failure (< 40%) due to prior myocardial infarction or any other cause; (3) cardiac arrest in the peri-intubation period; (4) known history of pulmonary arterial hypertension with or without right ventricular impairment; (5) moderate/severe known respiratory disease; (6) presence of left bundle branch block (LBBB); and (7) ICU admission due to massive pulmonary embolism (PE) confirmed by computed tomography pulmonary angiography (CTPA). Patients were also excluded if they presented signs of acute cor pulmonale and there was increased suspicion of massive PE and received thrombolysis (8). Poor acoustic window (9). patients with SARS-CoV-2 infection with a cause of ICU admission other than respiratory failure due to ARDS.

### Study protocol

All included patients had a full echocardiographic examination performed during the first 48 h of ICU admission. Patients were re-evaluated on the 10 ± 1 ICUday (per-protocol) and whenever necessary. During the echocardiographic examinations, the patients were ventilated according to the patients’ clinical status, respiratory drive, respiratory function.

### Measurements

(1) Patient characteristics and comorbidities, (2) Clinical data concerning ventilation/hemodynamics (3) Echocardiography data according to recent guidelines on conducting and reporting critical care echocardiography [20–25].

*Echocardiographic parameters:* Comprehensive transthoracic echocardiographic examination (System Vivid™ E95, GE Medical Systems, USA-Philips iE33, Philips Medical, USA) was performed to assess RV dimensions and function (2D/3D measurements) and the inferior vena cava (IVC) [21–25]. (Additional file 1).

Left ventricular systolic function was assessed using (1) the Simpson’s method to calculate ejection fraction estimation (2D) and (2) 3D left ventricular volume measurements. Both values 2D and 3D values are reported, as there were missing 3D values in some patients.

Right ventricular dilation was estimated through planimetry at end-diastole from a 4-chamber view quantification comparing the right ventricular end diastolic area (RVEDA) to left ventricular end diastolic area (LVEDA) to calculate their ratio (RVEDA/LVEDA). The RV contractility was estimated through measurements of the RV end-diastolic area (RVEDA) and end-systolic area (RVESA), measured to calculate RV Fractional Area Change (RVFAC % = 100 ×(RVEDA−RVESA)/RVEDA), Tricuspid Annular Plane Systolic Excursion (TAPSE), systolic velocity of the annulus of the tricuspid valve (RV S’) using tissue doppler imaging, while RV isovolumic acceleration (RV IVA) [derived from peak isovolumic velocity (IVV) and acceleration time (AT)] was also assessed. Two-dimensional speckle tracking echocardiography (2D-STE) was used to characterize longitudinal systolic strain [22]. RV longitudinal strain (RV–LS) was measured from the apical four-chamber view and the endocardial border was manually traced delineating a region of interest composed by 6 segments with eventual manual adjustments. Longitudinal strain curves were generated by the software for each RV segment. The RV free wall longitudinal strain (RV–LS) was calculated as the mean of the strain values in the three segments of the RV free wall [21, 25].

Right ventricular volumes and RV ejection fraction were estimated using three-dimensional echocardiography (3D). A wide-angled, single-beat, high frame rate (Heart Model mode) 3D full-volume images data sets were acquired from the apical four-chamber RV-focused view. Then, RV endocardial surfaces were defined and tracked throughout the cardiac cycle, and a quick minimal manual adjustment was performed in case of unsatisfactory outcomes. Finally, a 3D RV cast, RV volume curves were provided, from which the RV end-diastolic volume (RVEDV), RV end-systolic volume (RVESV), and RVEF were determined. All measures were made offline, using the semi-automated EchoPAC software package.

Right ventricular systolic pressure (RVSP) was estimated from peak tricuspid regurgitation (TR) jet velocity, using the simplified Bernoulli equation. Pulmonary artery systolic pressure (PASP) was estimated from the sum of RVSP plus the central venous pressure. Acute cor pulmonale (ACP) was defined if RVEDA/LVEDA was > 0.6 with presence of paradoxical septal motion.

Pulmonary vascular resistance (PVR) was indirectly estimated through quantification of the PASP (via tricuspid regurgitation velocity), the pulmonary acceleration time (PAcT) of the right ventricular outflow tract (RVOT) Flow velocity Doppler envelop, and the ratio of PASP to the RVOT velocity time (PASP/VTI_*RVOT)*_ as the ratio integrates PASP and cardiac output and thus better expresses changes in PVRs [26–28]. The presence of a systolic notch on the deceleration part was also reported [28]. Although the systolic notch was reported, it was not considered as an indication of increased PVRs, when calculating RV afterload. Right Ventriculoarterial Coupling, (VAC_*R*_) was assessed through the Tricuspid Annular Plane Systolic Excursion (TAPSE)/PASP ratio [10, 29].

Definitions for LV/RV impairments are presented in the Additional file 1.

The echocardiographic study was made by one operator (due to the pandemic conditions). Three consecutive cycles (five to ten in case of non-sinus rhythm) were averaged for every parameter. Measurements were assessed by three cardiologists (NK, VV and EZ) and trained doctors [competence in advanced critical care echocardiography (VT)]. Two of these doctors evaluated each measurement (offline using EchoPACK, or the stored videos). In case of > 10% variability, offline re-evaluation was performed with two operators present, to reach agreement.

#### Statistical analysis

Kolmogorov–Smirnov test was applied to test the variable distribution. Normally distributed variables were expressed as mean ± standard error of means (SEM), while non-normally distributed data were expressed as median (interquartile range); categorical variables were expressed as counts and percentages. The students *t* test or Wilcoxon rank-sum test were used where appropriate. Paired sample *t* test was used to compare variables between baseline and follow-up echocardiograms. Categorical variables were compared using the x^2^ test or Fisher exact test. Unadjusted correlations between TNI, LV/RV function indices, severity scores and respiratory variables were done using Pearson correlation.

Binary logistic regression analysis was used to investigate factors related to survival. Three models were constructed: in the first, baseline values that significantly differed between survivors/non-survivors were entered, in the second model, the significantly different echocardiographic values upon re-evaluation between survivors/non-survivors, and the third included the significantly different changes between survivors/non-survivors.

Reproducibility of echocardiographic measurements were tested on basic RV and LV indices. Interobserver agreement was assessed using interclass correlation coefficients.

Statistical analyses were performed using SPSS version 26.0 (IBM). A value of *p* < 0.05 (two-tailed) was considered statistically significant.

## Results

### Patients

**Fig. 1:**
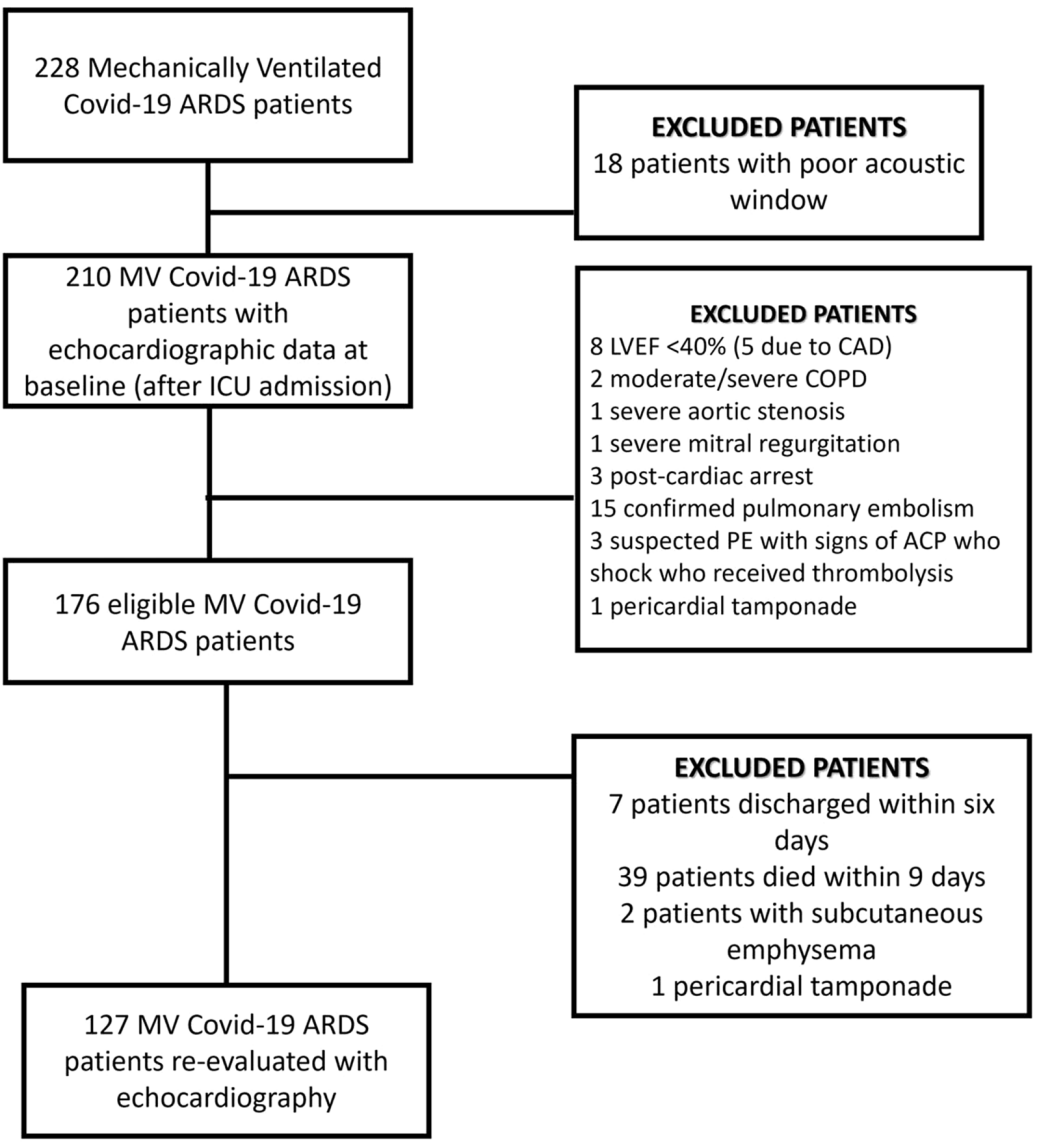
Flow chart. *ACP* acute cor pulmonale, *ARDS* acute respiratory distress syndrome, *CAD* coronary artery disease, *COPD* chronic obstructive pulmonary disease, *IC*U Intensive Care Unit, *LBBB* left bundle branch block, *LVEF* left ventricular ejection fraction, *MV* mechanical ventilation, *PE* pulmonary embolism;=

A total of 228 patients with COVID-19 ARDS were admitted, after intubation, in the ICU; 176 patients with echocardiography data upon ICU admission were analyzed (Fig. 1). Demographics, medical history and laboratory date upon ICU admission are presented in Additional file 1: Table S1. The mean age was 67.2 ± 0.8% and 65.8% were male.

### Respiratory and hemodynamic variables during initial echocardiography

At the time of the baseline echocardiographic evaluation, all patients were sedated and mechanically ventilated (Volume assist/controlled mode) and no patient presented spontaneous respiratory efforts. In 86.4% of the patients a neuromuscular blocking agent was administered. Variables concerning ventilatory parameters, respiratory variables and mechanics are presented in Table 1 (Additional file 1: Table S1).

**Table 1:** Respiratory, hemodynamic variables and respiratory mechanics during the initial echocardiographic evaluation

Clinical parameters	Data (number of patients with the reported value)
Demographics	
Age, years	67.2 ± 0.8 (176)
Sex (male), *n*, %	121/176 (65.8)
APACHE II score	16.86 ± 0.55 (176)
SOFA score	7.86 ± 0.16 (176)
Troponin, ng/ml (0–0.04)	0.34 ± 0.07 (176)
Respiratory variables	
Vt, ml/kg, (*n*)	6.9 ± 0.1 (176)
PEEP, cm H_2_O	11.7 ± 0.2 (176)
PaO_2_/FiO_2_, mm Hg	94.9 ± 3.8 (176)
PaCO_2_, mm Hg	53.5 ± 1.6 (176)
Hemodynamic Variables	
Noradrenaline, μg/kg/min	0.51 ± 0.07 (176)
MAP, mm Hg	68.53 ± 0.44 (176)
HR	76.07 ± 1.99 (176)
CVP, mmHg	12.60 ± 0.24 (158)
ScvO_2_, %	69.54 ± 0.52 (156)
Lac, mmol/Lt	3.4 ± 0.5 (148)
Respiratory System Mechanics	
C_*RS*_, ml/cm H_2_O	33.8 ± 0.9 (176)
P_*plat*_, cmH_2_O	25.9 ± 0.4 (176)
DP, cmH_2_O	14.3 ± 0.3 (176)

Numbers in parenthesis refer to the actual number of patients in whom the value was measured

*APACHE II* acute physiology and chronic health evaluation II score, *C*_*RS*_ static compliance of the respiratory system, *CVP* central venous pressure, *DP* driving pressure, *FiO*_*2*_ fraction of inspired oxygen, *HR* heart rate, *lac* lactate, *MAP* mean arterial pressure, *PaCO*_2_ partial pressure of arterial carbon dioxide, *PaO*_*2*_ partial pressure of arterial oxygen, *PEEP* positive end-expiratory pressure, *P*_*plat*_ plateau pressure, *ScvO*_*2*_ oxygen saturation in venous blood from vena cava, *SOFA score* sequential organ failure assessment score, *Vt* tidal volume

One hundred and seventy (96.6%) patients were receiving norepinephrine (mean dose 0.51 ± 0.09 μg/kg/min), while 19 patients (10.4%) were also receiving argipressin (Table 1). Upon admission, 86/176 (48.9%) presented elevated troponin levels [mean troponin 0.67 ± 0.13 ng/ml, in those with abnormal values (> 0.04 ng/ml)]. Age, BMI and sex did not differ between patients with increased and normal troponin levels (Additional file 1: Table S2).

#### Main echocardiographic findings

Seventeen patients presented atrial fibrillation (new onset atrial fibrillation in eight) at baseline evaluation; the rest were in sinus rhythm.

##### RV size/function

Values regarding RV size, RV function, IVC dimensions and distensibility index are presented in Table 2 and Additional file 1: Table S3. RV dilation was present in 151/176 (86%) [mean RV end-diastolic area/LV end-systolic area (RVEDA/LVEDA): 0.8 ± 0.02]. Acute cor pulmonale was present in 35 patients (20%). RVEDA/LVEDA correlated weakly to PaO_2_/FiO_2_ (r:− 0.168, *p* = 0.039) but not to PaCO_2_ values. RV systolic dysfunction (using at least two of the criteria mentioned in the Additional file 1) was present in 113/176 (64.2%) patients. RV dilation and dysfunction were simultaneously present in 93/173 (54%).

**Table 2:** Echocardiographic variables in survivors and non-survivors upon admission and follow-up

Echocardiographic Variable	COVID-19 ARDS survivors (*n* = 56)^*a*^			COVID-19 ARDS non-survivors (*n* = 120)		
	Admission	Day 10	*p* value	Admission	Day 10	*p* value
Age, years	65.9 ± 1.5			67.4 ± 1.0		
Sex, %	39 (69.6%)			80 (66.7%)		
APACHE II score	16.2 ± 0.9			17.3 ± 0.7		
SOFA score	7.5 ± 0.2			8.05 ± 0.2		
Right ventricle						
RVEDA/LVEDA	0.8 ± 0.05 (49)	0.6 ± 0.03 (49)#	0.001	0.81 ± 0.03 (78)	0.89 ± 0.03 (78)	0.864
ACP	12 (24.5)	5 (10.2)	0.073	21 (26.9)	14 (17.9)	0.850
RVEDA, cm^2^	22.1 ± 0.6 (49)	17.8 ± 0.8 (49)*	< 0.0001	23 ± 0.8 (78)	24.4 ± 0.8 (78)	0.097
RVESA, cm^2^	14.1 ± 0.5 (49)	10.3 ± 0.5 (49)#	< 0.0001	14.5 ± 0.6 (78)	15.2 ± 0.8 (78)	0.357
RV FAC, %	37.4 ± 1.6 (49)	41.1 ± 1.9 (49)	0.073	35.9 ± 1.6 (78)	38.9 ± 1.7 (78)	0.253
RVEDV^*b*^, ml	128.1 ± 13.9 (29)*	92.8 ± 8.4 (29)*	0.006	110.91 ± 6.02 (44)	121.3 ± 6.17 (44)	0.004
RVESV^*b*^, ml	82.4 ± 10.2 (29)*	52.2 ± 5.3 (29)*	0.002	67.6 ± 3.9 (44)	73.6 ± 4.4 (44)	0.127
RVEF^*b*^ (%)	36.5 ± 2.9 (29)	46.6 ± 2.1 (29)*	0.001	38.8 ± 1.5 (44)	39.1 ± 2 (44)	0.916
TAPSE, mm	19.8 ± 0.6 (51)	21.5 ± 0.5 (51)	0.008	20.3 ± 0.6 (73)	22 ± 2 (73)	0.404
RV S’, cm/sec	16.8 ± 0.8 (53)	19.9 ± 0.7 (53)#	0.002	15.3 ± 0.6 (74)	14.1 ± 0.5 (74)	0.045
RV IVA, m/sec^2^	3.98 ± 0.17 (53)	4.1 ± 0.17 (53)	0.318	3.66 ± 0.12 (74)	3.77 ± 0.14 (74)	0.260
RV–LS, %	− 13.6 ± 0.7 (42)	− 16.7 ± 0.8 (42)#	0.001	− 14.3 ± 0.8 (64)	− 12.6 ± 0.5 (64)	0.017
VTI_*RVOT,*_ cm	17 ± 0.7 (41)	19.4 ± 0.5 (41)#	0.011	16.2 ± 0.5 (60)	16.1 ± 0.4 (60)	0.987
PASP, mmHg	36.1 ± 2.4 (43)	20.1 ± 3 (43)#	< 0.0001	34.9 ± 1.8 (66)	36.3 ± 1.1 (66)	0.347
PASP/VTI_*LVOT*_, mmHg/cm	2 ± 0.2 (43)	0.9 ± 0.1 (43)#	< 0.0001	2 ± 0.2 (65)	1.9 ± 0.1 (65)	0.543
PASP/VTIR_*VOT*_, mmHg/cm	2.5 ± 1.4 (34)	1.1 ± 0.7 (34)#	< 0.0001	2.5 ± 1.5 (57)	2.4 ± 0.9 (57)	0.445
Pulmonary AcT, msec	61 ± 2.5 (41)	84.7 ± 2.4 (41)#	< 0.0001	66.8 ± 2.6 (60)	62.6 ± 2.3 (60)	0.096
RVOT notch	15 (23.4%)	4 (6.3%) *	0.033	21 (35%)	13 (21.6%)	0.321
VAC_R,_ mm/mmHg	0.80 ± 0.1 (43)	1.5 ± 0.1 (43)#	< 0.0001	0.73 ± 0.07 (60)	0.66 ± 0.08 (60)	0.497
Left ventricle						
LVEDD, mm	4.66 ± 0.1 (49)	4.62 ± 0.1 (49)	0.576	4.55 ± 0.06 (78)	4.53 ± 0.06 (78)	0.760
IVS, mm	0.9 ± 0.01 (49)			0.92 ± 0.01 (78)		
LVEDA, cm^2^	27.2 ± 1.03 (49)	27.1 ± 0.8 (49)	0.917	29.2 ± 0.7 (78)	27.9 ± 0.7 (78)	0.097
LVESA, cm^2^	17.4 ± 2.2 (49)	15.9 ± 1.5 (49)	0.555	16.3 ± 1.9 (78)	12.8 ± 1.2 (78)	0.357
VTI_*LVOT*_, cm	19.9 ± 0.1 (49)	21.8 ± 0.5 (49)*	0.005	20.2 ± 0.7 (78)	20.1 ± 0.5 (78)	0.914
LVEDV, ml (2D)	81.5 ± 4.5 (49)	85.9 ± 3.2 (49)	0.441	85.5 ± 4.9 (78)	80.7 ± 3.1 (78)	0.361
LVESV, ml (2D)	30.1 ± 2.5 (49)	32.1 ± 1.9 (49)	0.535	35.4 ± 3.8 (78)	32.5 ± 2 (78)	0.409
EF, %	62.5 ± 2.6 (49)	62.2 ± 2.8 (49)	0.932	60.4 ± 2.3 (78)	59.9 ± 1.7 (78)	0,869
SV, ml (Simpson’s)	50.1 ± 3.1 (49)	53.1 ± 2.7 (49)	0.600	47.6 ± 3.2 (78)	47.9 ± 2 (78)	0.754
LVEDV, ml (3D)^*b*^	82.5 ± 3.6 (30)	84.6 ± 2.8 (30)	0.553	82 ± 3.6 (42)	85.1 ± 3 (42)	0.187
LVESV, ml (3D)^*b*^	32.1 ± 1.9 (30)	34.5 ± 1.7 (30)	0.266	35 ± 2.2 (42)	35.3 ± 2 (42)	0.857
SV, ml (3D)^*b*^	50.3 ± 2.5 (30)	50.1 ± 2.5 (30)	0.952	46.9 ± 2.2 (42)	49.8 ± 2.1 (42)	0.221
EF (3D), %^*b*^	61.1 ± 1.5 (30)	58.6 ± 2.1 (30)	0.356	57.4 ± 1.6 (42)	58.4 ± 1.6 (42)	0.869
LV–LS, %	− 13.2 ± 0.8 (41)	− 17.4 ± 0.7 (41)#	< 0.0001	− 12.5 ± 0.6 (58)	− 11.9 ± 0.5 (58)	0.298
LV S’, cm/s	10.5 ± 0.8 (31)	10.6 ± 0.8 (31)	0.581	10.2 ± 0.6 (65)	10.4 ± 0.6 (65)	0.036
Pericardial effusion	17/56 (30.4%)*	14/49 (28.6%)*	1.000	53/120 (44.2%)	39/78 (50%)	1.000

Data are expressed as mean ± standard error of means

*A* left ventricular late diastolic filling velocity with atrial contraction, *ACP*, acute cor pulmonale, *E*, left ventricular early diastolic peak velocity, *E’*, early diastolic tissue Doppler velocity, *EF* ejection fraction, *LV–LS* longitudinal strain of the left ventricle, *IVA* isovolumic acceleration, *IVS* interventricular septum, *LVEDD* left ventricular end diastolic diameter, *LVEDV* left ventricular end diastolic volume, *LVESV* left ventricular end systolic volume, *LV s’* systolic tissue Doppler velocity measured at the lateral mitral annulus, *PASP* pulmonary artery systolic pressure, *PASP/VTI*_*RVOT*_ pulmonary artery systolic pressure to right ventricular outflow tract velocity time integral ratio, *RVEDA/LVEDA* right ventricular end diastolic area to left ventricular end diastolic area, *RVEDV* right ventricular end diastolic volume, *RVEF* right ventricular ejection fraction, *RVESV* right ventricular end systolic volume, *RVFAC* right ventricular fractional area change, *RV–LS* right ventricular free wall longitudinal strain, *RV S’* systolic tissue Doppler velocity measured at the lateral tricuspid annulus, *RV SV* right ventricular stroke volume, *SV* stroke volume, *TAPSE* tricuspid annular plane systolic excursion, *VAC*_*R*_ right ventricular to pulmonary artery coupling, *VTI*_*LVOT*_, left ventricular outflow tract velocity time integral, *VTI*_*RVOT,*_ right ventricular outflow tract velocity time integral

^a^Numbers in parenthesis refer to the actual number of patients that the variable was measured

^b^3D measurements

Right ventricular longitudinal free wall strain (RV–LS) was severely reduced [RV–LS − 14.3 ± 0.4%. RV–LS > − 20% was present in almost the whole cohort 131/145 (90.3%) patients measured], while RV–LS ≥ − 17 (average value reported in severe COVID-19 patients) was present in 90/145 (62%) [30].

##### RV afterload

Tricuspid regurgitation could be measured in 150 patients, VTI_*RVOT*_ in 142 and VTI_*LVOT*_ in 176 patients. Data on PVRs are presented in Table 2 and Additional file 1: Table S3. Increased PVRs were present in 118/150 (78.7%) of the patients using at least one variable from the definition used. PASP > 38 mmHg was present in 44/150 (29.3%) patients, PAcT < 90 ms in 110/142 (77.5%), PASP/VTI_*RVOT*_ > 2 in 71/128 (55.5%), while a mid-systolic notch in the RVOT pulse wave doppler signal (not included in increased PVR definition) was present in 36/142 (25.4%) patients. PASP/VTI_*RVOT*_ correlated with TAPSE: − 0.349, *p* < 0.0001, RV–EF: − 0.277, *p* = 0.026, PAcT: − 0.296, *p* = 0.001 and VAC_*R*_: − 0.523, *p* < 0.0001.

The right ventriculoarterial coupling (VAC_R_) was impaired (0.8 ± 0.06 mm/mmHg).

Pericardial effusion was present in 70/176 (39.7%) of the patients. In the majority (68/70) the effusion was mild (< 10 mm) (in diastole).

##### LV function

Mean EF was nearly normal (57.8 ± 1.1%); LV systolic dysfunction (LVEF < 40%) was present in 21/176 (11.9%), while severely decreased (EF < 30%) in 7 patients (4%). None of these patients had any known history of LV cardiomyopathy. However, LV–LS was reduced (− 13.3 ± 0.3%), 127/145 (87.5%) patients presented LV–LS > − 18%, while in 82/145 (56.5%) LV–LS was > − 15.9% (Lower Limit Normal) and > − 14 in 67 (46.2%) (Additional file 1: Table S3).

### Echocardiographic time course of COVID-19 cardiac involvement

Echocardiographic re-evaluation upon the 10th ICUday was performed in 127 patients (Fig. 1). Thirty-nine patients had died (day-10 non-survivors). Ten-day non-survivors presented worse respiratory system mechanics and a trend for worse hemodynamics compared to baseline values (Additional file 1: Table S4). Compared to day-10 survivors, they presented higher troponin levels upon ICU admission (0.78 ± 0.19 vs 0.21 ± 0.06 ng/ml, *p* < 0.0001), higher prevalence of pericardial effusion [22/39 (56.4%) vs 48/137 (35%), *p* = 0.015] and lower RVFAC (31.7 ± 2% vs 37.7 ± 1%, *p* = 0.011) (Additional file 1: Table S4).

**Fig. 2:**
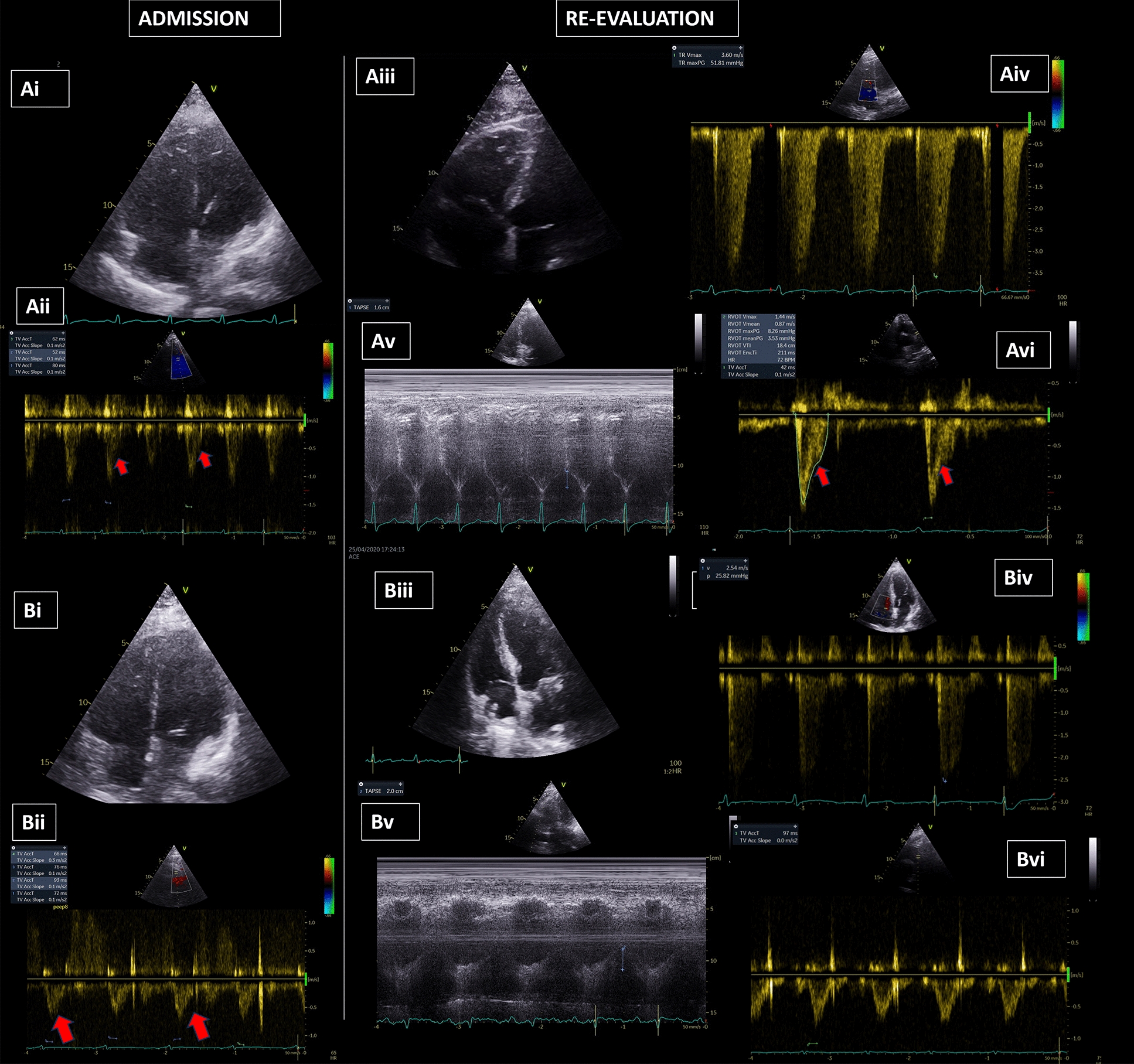
Echocardiographic findings in patients with COVID-19 ARDS. **A** Non-survivor patient. (Αi) non-survivor with RV dilation on ICU admission; (Aii) PAcT: 64.6 ms Red arrows indicate the early systolic notch in the ascending part of the RVOT envelope. Please not the triangular spahe of the RVOT envelope indicating increased PVRs. (Aiii) RV dilatation upon re-evaluation; (Aiv) TRV: 3.6 m/sec ≥ PASP = 51.81 mmHg + (CVP: 13 mmHg) = 64.25 mmHg (Av) TAPSE: 1.6 cm (re-evaluation); (Avi) PAcT: 42 ms Red arrows indicate the early systolic notch in the ascending part of the RVOT envelope. Please not the triangular spahe of the RVOT envelope indicating increased PVRs. **B** Survivor patient. (Bi) survivor with RV dilatation upon ICU admission; (Bii) PAcT: 76.75 ms Red arrows indicate the early systolic notch in the ascending part of the RVOT envelope. Please not the triangular spahe of the RVOT envelope indicating increased PVRs, (Biii) normal size of the RV upon re-evaluation; (Biv) TRV: 2.54 m/sec ≥ PASP = 25.82 mmHg + (CVP: 8 mmHg) = 33.82 mmHg (Bv) TAPSE: 2 cm; (Bvi) PAcT: 95 ms, Please not the normal parabolic shape of the RVOT envelope in indicating normal PVRs. *ARDS* acute respiratory distress syndrome, *CVP* central venous pressure, *COVID-19* coronavirus disease 2019, *PASP* pulmonary artery systolic pressure, *PVR* pulmonary vascular resistance, *PAcT* pulmonary acceleration time, *RV* right ventricle, *TAPSE* tricuspid annular plane systolic excursion, *TRV* tricuspid regurgitation velocity

In the rest 127 patients, RV systolic function (RVFAC: 36.6 ± 0.9 to 39.1 ± 0.1, *p* = 0.048), and RV afterload (PASP/VTI_*RVOT:*_ 2.29 ± 1.4 to 1.9 ± 0.1, *p* < 0.0001 and PAcT: 65.3 ± 1.5 to 72 ± 2.1, *p* = 0.035), presented significant improvements (Additional file 1: Table S3, Fig. 2). Isovolumic Acceleration of the RV (RV IVA) was normal and did not change upon re-evaluation.

### Troponin

Upon admission, 86/176 (48.9%) presented elevated troponin levels [mean troponin 0.67 ± 0.13 ng/ml, in those with abnormal values (> 0.04 ng/ml)]. Age, BMI and sex did not differ between patients with increased and normal troponin levels (Additional file 1: Table S2).

The RV was more dilated (RVEDA/LVEDA: 0.85 ± 0.04 vs 0.79 ± 0.04, *p* = 0.037) in patients with higher troponin. Troponin levels were not correlated with RVEF or RV–LS. On the contrary, troponin presented a positive correlation to the presence of pericardial effusion (r:0.293, *p* < 0.0001).

### Outcome

Patients were followed up until ICU discharge. The survival rate in the present cohort was 34.8%. Oxygenation did not differ (PaO_2_/FiO_2_: 86.3 ± 5.2 vs 95.9 ± 4.7 mmHg, *p* = 0.196), but survivors had better lung mechanics (Pplat: 23.9 ± 0.5 vs 27.3 ± 0.4 cmH_2_O, *p* < 0.0001, driving pressure (DP): 12.3 ± 0.4 vs 15.4 ± 0.5 cmH_2_O, *p* < 0.0001, respiratory system compliance (C_*RS*_): 38.7 ± 1.5 vs 31 ± 1.1 ml/cmH_2_O, *p* < 0.0001) (Additional file 1: Table S5) upon admission. Survivors also presented lower troponin levels at baseline (0.05 ± 0.01 vs 0.49 ± 0.1 ng/ml, *p* = 0.001).

Respiratory variables upon re-evaluation were significantly different in the two subgroups (survivors vs non-survivors) (Table 3). Survivors presented significant improvements in PaO_2_/FiO_2_, 231.2 ± 11.9 vs 120.2 ± 6.7 mmHg; PaCO_2_, 43.1 ± 1.2 vs 53.9 ± 1.5 mmHg; respiratory system compliance—C_*RS*_, 42.6 ± 2.2 vs 27.8 ± 0.9 ml/cmH_2_O, all *p* < 0.0001. Echocardiographic variables in survivors/non-survivors are presented in Table 2. Baseline echocardiographic data did not differ between survivors/non-survivors, apart from a higher incidence of pericardial effusion in non-survivors (30.4% vs 44.2%, *p* = 0.019). In survivors only, a decrease in RV afterload was noted (PASP: 36.1 ± 2.4 to 20.1 ± 3 mmHg, *p* < 0.0001, PASP/VTI_*RVOT*_: 2.5 ± 1.4 to 1.1 ± 0.7, *p* < 0.0001, PAcT: 61 ± 2.5 to 84.7 ± 2.4 ms, *p* < 0.0001), associated with RV systolic function improvement (RVEF: 36.5 ± 2.9% to 46.6 ± 2.1%, *p* = 0.001 and RV–LS: − 13.6 ± 0.7% to − 16.7 ± 0.8%, *p* = 0.001) In addition, RV dilation subsided in survivors (RVEDA/LVEDA: 0.8 ± 0.05 to 0.6 ± 0.03, *p* = 0.001). Right ventriculoarterial coupling improved significantly in survivors only (VAC_*R*_: 0.8 ± 0.1 to 1.5 ± 0.1, *p* < 0.0001), while in non survivors further worsened (VAC_*R*_: 0.73 ± 0.07 to 0.66 ± 0.08, *p* < 0.0001). Survivors presented significant improvements in LV systolic function as depicted by LV–LS (− 17.4 ± 0.7% vs − 13.2 ± 0.8%, *p* < 0.0001) compared to non-survivors (− 11.9 ± 0.5% vs − 12.5 ± 0.76%, *p* = 0.298). Respiratory variables upon re-evaluation correlated with RV function and afterload (Additional file 1).

**Table 3:** Respiratory variables between survivors and non-survivors upon echocardiographic re-evaluation

	Survivors	Non-survivors	*p* value
Mode (volume control/pressure support)	16/33	75/3	< 0.0001
PEEP, cmH_2_O	7.9 ± 0.4	11.1 ± 0.4	< 0.0001
Driving pressure, cmH_2_O	11 ± 0.4	15 ± 0.3	< 0.0001
C_*RS*_, ml/cmH_2_O	42.6 ± 2.2	27.8 ± 0.9	< 0.0001
PaO_2_/FiO_2_, mmHg	231.2 ± 11.9	120.2 ± 6.7	< 0.0001
PaCO_2_, mmHg	43.1 ± 1.2	53.9 ± 1.5	< 0.0001
pH	7.43 ± 0.01	7.26 ± 0.01	< 0.0001
Noradrenaline, μcg/kg/min	0.06 ± 0.02	0.46 ± 0.05	< 0.0001

*C*_*RS*_ respiratory system compliance, *PaCO*_*2*_ partial carbon dioxide pressure, *PaO*_*2*_*/FiO*_*2*_ partial oxygen pressure/fraction of inspired oxygen, *PEEP* positive end expiratory pressure

Three multivariable regression models were constructed to identify values associated with mortality. In the first, baseline values that significantly differed between survivors/non-survivors were entered and revealed that only C_*RS*_ (OR 0.842 95%CI 0.721–0.982, *p* = 0.028) and Pplateau (OR 1.425 95%CI 1.024–1.982, *p* = 0.036) were independent factors. In the second model, the significantly different echocardiographic values upon re-evaluation were tested, revealing D-10 LV–LS (OR 1.881 95%CI 1.105–3.203, *p* = 0.020), while in the third model ΔPASP/VTI_*RVOT*_ (OR 78.269 95%CI 2.578–2376.236, *p* = 0.021) and ΔRV–LS (OR 0.032 95%CI 0.001–0.908, *p* = 0.044) were independently associated with mortality (Additional file 1: Table S6).

#### Interobserver variability

In case of disagreement in measurements (> 10% variability) re-evaluation was performed with all operators present, to reach agreement. Interobserver agreement (in the captured echocardiographic measurements) was high [interclass correlation coefficients for different indices were: RV FAC: 0.955, TAPSE: 0.960, RV–LS: 0.957, RV EF: 0.919, PASP: 0.966, VTI_*RVOT*_: 0.988, VTI_*LVOT*_: 0.942, LV EF(2D): 0.979; LV EF(3D): 0.934]. Bland and Altman plots are presented in Additional file 1: Table S7.

## Discussion

To our knowledge, this is the first study to comprehensively evaluate the temporal course of cardiac impairment with echocardiography in a large cohort of mechanically ventilated COVID-19 ARDS patients and investigate its implications in survival. COVID-19. The results indicate the presence of significant RV dilation and systolic dysfunction, accompanied by increased pulmonary vascular resistances. Although baseline echocardiographic data did not differ between survivors and non-survivors, the right ventricular size, function and afterload improved only in survivors after a 10-day period, and so did both LV and the RV longitudinal strain. In fact, the improvements in RV afterload were associated with RV systolic function improvements and survival. Finally, the respiratory system function upon re-evaluation correlated with RV function and afterload.

### RV function–RV afterload

During the pandemic, the RV has been extensively identified as the most frequent cardiovascular target in COVID-19 [7, 8, 30]. To be more strict, we defined RV systolic dysfunction when there were at least two indices indicating systolic impairment. Both RV dilation and dysfunction were present in 56% of the patients. RV–LS was severely impaired (− 14.4%) and RV–LS > − 20% was present in 91% of the patients, corroborating previously reported data in small cohorts of mixed (ICU and non-ICU) COVID-19 patients [31–33]. Even using more strict criteria (RV–LS > − 17% as the reference value, reported in COVID-19 patients [30]) impaired strain was observed in 62% of the patients. Current data in COVID-19 report a greater RV strain impairment in the more severe patients. RV–LS has been reported to have higher values (more impaired) in ARDS than non-ARDS patients (− 21.3% vs. − 24.6%), in ICU than non-ICU patients (− 17.5% vs − 19.8%) and in non-survivors than survivors (− 14% vs − 19%) [30, 34–36]. However, the MV settings and respiratory system mechanics are not reported, thus, one cannot conclude on the effects of MV on the observed results. RV involvement in COVID-19 ARDS seems to be related to the increased afterload due to COVID-19-induced microthrombosis, lung mechanics’ impairment and mechanical ventilator settings, which may have additional contribution, as we have recently shown [37]. Surprisingly, RV IVA, a load independent variable indicating RV systolic dysfunction, was normal during the two timepoints of measurements, supporting the role of increased RV afterload in RV dysfunction.

In our cohort, baseline echocardiographic values concerning the RV did not differ between survivors and non-survivors. On the contrary, re-evaluation during the first 10 days of ICU stay revealed a reversible impairment of RV dilation, systolic dysfunction and afterload in survivors. Moreover, ΔPASP/VTI_*RVOT*_ marker of RV afterload, was associated with survival. This might reflect the improvement in the RV afterload burden, resulting from improvements in ARDS, respiratory system mechanics and vascular obstruction, thus decreasing the amplitude of positive pressure (PEEP) requirements, as indicated in the respiratory variable differences between survivors and non-survivors upon re-evaluation. Right ventricular failure development during the course of ICU stay was associated with worsening in respiratory system physiology (oxygenation, ventilatory ratio and driving pressure) in a recent study presenting echocardiographic data in COVID-19 ARDS patients, further supporting the importance of RV afterload in the myocardial performance of the RV [13]. Right ventricular failure was ultimately associated with mortality [13]. On the other hand, direct myocardial inflammation attenuation cannot be excluded. Moreover, we cannot conclude whether the lack of RV improvement in non-survivors might also present a marker of septic cardiomyopathy presenting upon the 10th ICUday, as sepsis was more frequent in this subset of patients. RV dysfunction is a common finding in early sepsis [37].

### Right ventricular–arterial coupling (VACR)

The present study depicts the uncoupling between RV contractility and afterload in MV COVID-19 ARDS patients. In our cohort, ventriculoarterial uncoupling was equally impaired in survivors vs non-survivors. Only in survivors did VAC_*R*_ improve, accompanying possibly the improvements in RV systolic function and the decrease in RV afterload, while in non-survivors it further deteriorated (Table 2). Early and pronounced RV–PA uncoupling has been recently reported in COVID-19 ARDS patients; survivors presented a TAPSE/PASP of 0.89 ± 0.29 vs 0.51 ± 0.22 mm/mmHg found in non-survivors; again the information on lung mechanics is missing [10]. Under this perspective, we highlight the interplay between respiratory system function, RV function and afterload. Herein, we show that the careful evaluation of RV myocardial performance in relation to RV afterload, affected by respiratory physiology and underlined by a means of measuring RV–PA coupling (herein assessed through TAPSE/PASP) is of great significance to assess patient outcomes.

### LV function

Using conventional echocardiographic measurements, LV function was within normal levels in most patients; EF < 40% was present in 11.9% of the patients. In a multicenter study across European ICUs, using conventional echocardiography, LV systolic dysfunction was found in 22% of the patients (69% mechanically ventilated), 30% of whom had a previous history of cardiomyopathy [9]. In our study, we excluded patients with pre-existing left ventricular dysfunction, so that the findings could more clearly depict the impact of COVID-19 in cardiac function. In accordance with our results, Doyen et al. found that among 30 MV COVID-19 ARDS patients, LV systolic dysfunction was present in 13% [38].

Interestingly, speckle tracking echocardiography revealed a “silent” impairment in 87.5% of the intubated COVID-19 patients, with a mean LV–LS of − 13.3 ± 0.3%. Various studies have focused on LV–LS in COVID-19 patients, ranging between − 17.9% and (− 18.4%), but included patients of different illness severity, usually spontaneously breathing and only a minority included MV patients [35, 39]. In our study, including the most severe ARDS patients, with a mean PaO_2_/FiO_2_ of 94.9 mmHg under MV, with the majority (91.5%) requiring vasopressors, LV–LS was lower and probably reflected the true myocardial dysfunction, not revealed with LVEF, a load dependent parameter. This discrepancy between LVEF and LV–LS was recently pointed in a cohort of mixed severity COVID-19 patients [35]. Janus et al. reported a mean LV–LS of − 11.8% in 31 patients, yet there are no data concerning pneumonia severity and oxygenation impairment [40]. To our knowledge, our study is the first to report results on strain imaging in a homogenous population of MV COVID-19 ARDS patients. Moreover, contradicting previous findings reporting the ability of LV–LS to predict survival in mixed severity cohorts [36, 40], we found that only LV–LS upon re-evaluation, along with PASP/LVOT_*VTI*_, were independently associated with mortality in MV patients.

### Troponin

Troponin levels have been used to indicate myocardial inflammation in COVID-19 patients. [41]. Yet, TNI was measured when clinically indicated, thus, the correlation of TNI to the presence of RV/LV dysfunction might encounter a selection bias. Subsequent scarce echocardiographic data have reported conflicting results concerning the correlation to cardiac function impairment [32, 42, 43]. In our study, troponin was increased in 47.3% of the patients. TNI levels were higher in patients with greater RV dilation but, troponin did not correlate to RV dysfunction indices. Jansson et all found that acute myocardial injury, occurred in 82% ICU COVID-19 ARDS patients, yet troponin did not correlate with RV/LV impairment [43, 44]. Similarly, Karagodin et al., in the global WASE COVID-19 study, found that troponin was increased in 35% of the patients included, contrasting the overall good RV and LV function [36]. Indeed, increased troponin in critical illness and sepsis is multifactorial and may not result from direct myocardial necrosis; [45, 46]. Thus, troponin levels might serve as a primary marker of illness severity and second, reflect direct myocardial damage.

Moreover, we found an increased incidence of pericardial effusion supporting previous data [38]. Pericardial effusion presents a direct sign of cardiac involvement, although not warranting intervention, in the majority of the patients. It may also indicate severity of infection, as a higher incidence has been reported in ICU vs non-ICU patients (23.2% vs 16.3%) [36]. Pericardial effusions and higher troponin were more frequently found in non-survivors, indicating probably that they suffered a direct myocardial COVID-19 impairment. Troponin levels and pericarditis might indicate disease severity, not depicted by usual scores (SOFA, APACHE II).

The study’s monocentric character is a certain limitation. Yet, a large number of consecutive intubated patients with severe ARDS underwent a comprehensive echocardiographic evaluation on ICU admission, while echocardiographic data on the time course of cardiac function are also presented. The selection of the re-evaluation timepoint, although seems arbitrary at first sight, was based on the median intubation time of 10–14 days reported in large-scale observational studies [14–17]. Moreover, the re-evaluation echocardiographic study was performed under different conditions as many patients were receiving less sedation and were on a spontaneous mode; thus, we do not discuss on IVC distensibility differences between the two timepoints. The cohort presented increased mortality (68.2%); the patients were admitted only after intubation, thus we included a cohort with a rather increased illness severity. In fact, disease severity scores were higher in our cohort compared to other studies reporting mortality rates between 35% and 50.6% [14–17]. Among the most severe patients, the mean case fatality rate has been found around 45% in patients receiving invasive mechanical ventilation, while a case fatality rate exceeding 78%, has also been noted, depending on the timing of intubation, time with respiratory distress or other regional disparities [17, 22, 27, 47]. Many patients were intubated “late”, a factor found to impact lung mechanics and survival [48], while the immunomodulatory treatments that the patients received might have also affected the outcome, but this issue is beyond the scope of the present study. Finally, the exact interobserver variability cannot be calculated for every measurement, as per protocol, we re-evaluated the measurements with > 10% variability with all operators present to reach agreement. Moreover, only one operator at a time performed each echocardiographic study, due to the pandemic’s nature.

## Conclusion

The study confirms that the myocardial function, especially the right ventricle, is affected in MV COVID-19 ARDS patients. COVID-19 improvements in RV function, RV afterload and RV–PA coupling at day 10 were associated with respiratory function improvements and survival. Further multicentric study is needed to confirm these findings and assess the therapeutic and prognostic impact of serial comprehensive echocardiography.

### Supplementary Information

**Additional file 1: Table S1.** Demographics in the whole cohort. **Table S2.** Demographic data and respiratory variables in COVID-19 ARDS patients according to the presence of increased troponin levels. **Table S3.** Echocardiographic variables in the whole cohort upon ICU admission and upon the 10th ICU day. **Table S4.** Clinical data and echocardiographic variables of initial evaluation between 10-day survivors and non-survivors. **Table S5.** Baseline characteristics and outcome between survivors and non-survivors. **Table S6.** Univariate and multivariate regression models to identify predictors of survival. **Table S7.** Bland and Aldman scatter plots to estimate interobserver variability for different RV measurements. **Table S8.** Clinical characteristics and echocardiographic variables in patients stratified according to the troponin value upon admission. Echocardiographic data upon re-evaluation on the 10th ICU day.

## Data Availability

The data sets will be available from the corresponding author upon reasonable request.

## Abbreviations

AcT: Acceleration time
ANOVA: Analysis of variance
ARDS: Acute respiratory distress syndrome
AUC: Area under the curve
BMI: Body mass index
CI: Confidence interval
CVP: Central venous pressure
COVID-19: Coronavirus disease 2019
C_*RS*_: Static compliance of the respiratory system
ΔIVC: Respiratory variability in Inferior Vena Cava diameter [(IVCmax–IVCmin)/IVCmin]
DP: Driving pressure
E: Left ventricular early diastolic peak velocity
E’: Early diastolic tissue Doppler velocity
EF: Ejection fraction
FiO_2_: Fraction of inspired oxygen
ICU: Intensive Care Unit
IVC: Inferior vena cava
lac: Lactate
LBBB: Left bundle brunch block
LV: Left ventricle
LVOT: Left ventricular outflow tract
LV–LS: Longitudinal strain of the left ventricle
LV S’: Systolic tissue Doppler velocity measured at the lateral mitral annulus
MAP: Mean arterial pressure
MV: Mechanical ventilation
PaCO_2_: Partial pressure of arterial carbon dioxide
PaO_2_: Partial pressure of arterial oxygen
PASP: Pulmonary artery systolic pressure
PASP/VTI_*LVOT*_: Pulmonary artery systolic pressure to left ventricular outflow tract velocity time integral ratio
PASP/VTI_*RVOT*_: Pulmonary artery systolic pressure to right ventricular outflow tract velocity time integral ratio
PEEP: Positive end expiratory pressure
P_*pl*_: Pleural pressure
P_*plat*_: Plateau pressure
PPV: Pulse pressure variation
PVR: Pulmonary vascular resistance
RV: Right ventricle
ROC: Receiver operating characteristics
RVEDA/LVEDA: Right ventricular end diastolic area to left ventricular end diastolic area
RVEDV: Right ventricular end diastolic volume
RVESV: Right ventricular end systolic volume
RVEF: Right ventricular ejection fraction
RVFAC: Right ventricular fractional area change
RVOT: Right ventricular outflow tract
RV–LS: Right ventricular longitudinal strain
RV S’: Systolic tissue Doppler velocity measured at the lateral tricuspid annulus
SEM: Standard error of means
ScvO_2_: Oxygen saturation in venous blood from vena cava
SV: Stroke volume
TAPSE: Tricuspid annular plane systolic excursion
VAC_*R*_: Ventriculoarterial coupling of the right ventricle to the pulmonary artery
Vt: Tidal volume
VTI_*LVOT*_: Left ventricular outflow tract velocity time integral
VTI_*RVOT*_: Right ventricular outflow tract velocity time integral
SARS-COV-2: Severe acute respiratory syndrome coronavirus 2
