# Supplementary material for: Comprehensive temporal analysis of right ventricular function and pulmonary haemodynamics in mechanically ventilated COVID-19 ARDS patients
Source: Ann Intensive Care. 2024 Feb 12;14:25. doi: 10.1186/s13613-024-01241-1 (PMC10861421; doi:10.1186/s13613-024-01241-1)
Supplement: Supplementary file 1 — Additional file 1: Table S1. Demographics in the whole cohort. Table S2. Demographic data and respiratory variables in COVID-19 ARDS patients according to the presence of increased troponin levels. Table S3. Echocardiographic variables in the whole cohort upon ICU admission and upon the 10th ICU day. Table S4. Clinical data and echocardiographic variables of initial evaluation between 10-day survivors and non-survivors. Table S5. Baseline characteristics and outcome between survivors and non-survivors. Table S6. Univariate and multivariate regression models to identify predictors of survival. Table S7. Bland and Aldman scatter plots to estimate interobserver variability for different RV measurements. Table S8. Clinical characteristics and echocardiographic variables in patients stratified according to the troponin value upon admission. Echocardiographic data upon re-evaluation on the 10th ICU day. [file 13613_2024_1241_MOESM1_ESM.docx]

**Additional file**

**Comprehensive temporal analysis of right ventricular function and pulmonary haemodynamics in Mechanically ventilated Covid-19 ARDS patients.**

**Vasiliki Tsolaki^1^, George E Zakynthinos^2^, Nikitas Karavidas^1^, Vasileios Vazgiourakis^1^, John Papanikolaou^3^, Kyriaki Parisi^1^, Paris Zygoulis^1^, Demosthenes Makris^1^ and Epaminondas Zakynthinos^1^**

1. Critical Care Department, University Hospital of Larissa, University of Thessaly, Faculty of Medicine, Mezourlo 41110, Larissa, Greece
2. Department of Cardiology, General Hospital of Trikala, Karditsis 56, 42131 Trikala, Thessaly, Greece.
3. Department of Emergency Medicine, General University Hospital of Larissa, University of Thessaly, Faculty of Medicine, Mezourlo 41110, Larissa, Greece.

**Running Title:** Cardiac function in intubated Covid-19 ARDS patients.

**Corresponding author:**

Epaminondas Zakynthinos, Professor of Critical Care Medicine

Critical Care Department, General University Hospital of Larissa, University of Thessaly, Faculty of Medicine, Mezourlo 41110, Larissa, Greece [ezakynth@yahoo.com](mailto:ezakynth@yahoo.com), tel 0030241352013, fax 2413501018

**METHODS**

**Echocardiography**

Comprehensive transthoracic echocardiographic examinations were performed with two devices: System Vivid^TM^ E95, GE Medical Systems, USA and Philips iE33, Philips Medical USA.

Covid-19 patients in our department are hospitalized in two distinct departments. Each department has one echocardiographic device (GE and Phillips IE33). The machines were not transferred from one unit to the other, but the personnel acquiring the images had access to both units. Strain and 3D measurements were only performed in patients hospitalized in the department with the GE device (providing the software for strain and 3D measurements). Apical long axis (four and two chamber) clips obtained with a frame rate >50 Hz underwent off-line speckle tracking analyses on the semi-automated EchoPAC package (GEMS).

Left ventricular systolic function was assessed using (1) the Simpson’s method to calculate ejection fraction estimation (2D) and (2) 3D left ventricular volume measurements. Both values 2D and 3D values are reported, as there were missing 3D values in some patients.

Right Ventricular dilation was estimated through planimetry at end-diastole from a 4-chamber view quantification comparing the Right Ventricular End Diastolic Area (RVEDA) to Left ventricular End Diastolic Area (LVEDA) to calculate their ratio (RVEDA/LVEDA). The RV contractility was estimated through measurements of the RV end-diastolic area (RVEDA) and end-systolic area (RVESA), measured to calculate RV Fractional Area Change (RVFAC %=100x(RVEDA-RVESA)/RVEDA), Tricuspid Annular Plane Systolic Excursion (TAPSE), systolic velocity of the annulus of the tricuspid valve (RV S’) using tissue doppler imaging and two-dimension Speckle Tracking Echocardiography (2D-STE) to characterize longitudinal systolic strain [1]. RV Longitudinal Strain (RV-LS) was measured from the apical 4-chamber view and the endocardial border was manually traced delineating a region of interest composed by 6 segments with eventual manual adjustments. Longitudinal strain curves were generated by the software for each RV segment. The RV Free Wall Longitudinal Strain (RV-LS) was calculated as the mean of the strain values in the three segments of the RV free wall [2,3].

Right Ventricular volumes and RV ejection fraction were estimated using three-dimension echocardiography (3D). A wide-angled, single-beat, high frame rate (Heart Model mode) 3D full-volume images data sets were acquired from the apical 4- chamber RV-focused view. The 3DE datasets were stored digitally for offline analysis. The 3D full-volume RV images were analyzed by four experienced echocardiographers (EZ, NK, VV, VT – at least two operators present for each measurement). RV-focused one-beat, 3D full-volume images were analyzed with a novel, full automated RV quantification software (3D Auto RV, version 203 GE Vingmed Ultrasound AS) that detect RV endocardial contours using artificial intelligence, which consists of knowledge-based identification of initial global shape and RV chamber orientation. The software initially identified LV and RV long axis landmarks at end-diastole in the apical two- and four chamber views. Based on that, the RV-focused four-chamber view and a short-axis view were constructed. Then RV endocardial surfaces were full automatically defined and tracked throughout the cardiac cycle, and a quick minimal manual adjustment was performed in case of unsatisfactory outcomes. Finally, a 3D RV cast, RV volume curves were provided, from which the RV end-diastolic volume (RVEDV), RV end-systolic volume (RVESV), and RVEF were determined. All measures were made offline, using the semi-automated EchoPAC software package.

Right Ventricular Systolic Pressure was estimated from peak Tricuspid Regurgitation (TR) jet velocity, using the simplified Bernoulli equation, combining this value with an estimate of the RA pressure [determined by the Central Venous Pressure at end expiration]: RVSP = 4(V)^2^ + RA pressure. The CVP was estimated as CVP_measured_ – 1/3 PEEP value. For patients with suboptimal TR jet, optimization was attempted through normal saline babble infusion. Pulmonary Vascular Resistance was indirectly estimated through quantification of the Right Ventricular Systolic pressure, the Acceleration Time (AcT) and the presence of a systolic notch on the deceleration part of the Right Ventricular Outflow Tract Flow Velocity Doppler envelope [4]. Although the systolic notch was reported, it was not considered as an indication of increased PVRs, when calculating RV afterload. We calculated the ratio of Pulmonary Artery Systolic Pressure to Velocity Time Integral of the Left Ventricular Outflow Tract (PASP/VTI*_LVOT_*) as a surrogate to roughly indicate Pulmonary Vascular resistance. To investigate whether the two derived values from VTI*_LVOT_* and VTI*_RVOT_* are correlated we also measured PASP/VTI*_RVOT_* as the measurement of RVOT*_VTI_* is sometimes not feasible in patients using increased PEEP values [5,6].

The coupling of the RV to the pulmonary circulation (Right Ventriculoarterial Coupling, VAC*_R_* was also assessed. For this purpose, VAC*_R_* was estimated as the TAPSE/PASP ratio, previously shown to be a valid surrogate of the gold standard ratio of end-systolic to arterial elastance (Ees/Ea) for the assessment of RV-arterial coupling [7].

Areas of the left and right ventricle, TAPSE, tricuspid regurgitation and the mitral inflow and Tissue Doppler Imaging derived measures were obtained from the apical 4-chamber view, while VTI*_LVOT_* was obtained through an apical five chamber view. For TR measurement, the modified parasternal view was also used. VTI*_RVOT_*  was measured from the parasternal view.

Inferior Vena Cava (IVC) diameter and respiratory variability [(IVCmax-IVCmin)/IVCmin] was determined through the subcostal window [1].

Each echocardiographic study was performed by one doctor. Measurements were done offline (EchoPAC) by two doctors among three cardiologists (NK, VV and EZ) and one trained doctor [competence in advanced critical care echocardiography (VT). In case of disagreement (>10% variability) re-evaluation was performed with all operators present, to reach agreement.

**Definitions:**

*Left ventricular dysfunction* was defined as an EF<40% (severe <30%) and/or LV-LS>-18% [1,2].

*Right Ventricular dilation* was present when RV End Diastolic Area/LV End Diastolic Area (RVEDA/LVEDA) >0.6 [1].

*Right ventricular dysfunction* was considered if at least two of the following indices were present: fractional area change (RVFAC)≤35%, RV tissue doppler systolic excursion (RV S’) ≤10 cm/sec, TAPSE≤16 mm, RV longitudinal strain (RV-LS) was >-20% or RVEF <44% (using 3D echocardiography) [1]. Systolic RV function is altered when Isovolumic Acceleration (IVA) is below 2.2 m/sec².

*Increased pulmonary afterload* was present if the patients presented at least one of the following: Pulmonary Artery Systolic Pressure (PASP)>38 mmHg, Pulmonary Acceleration Time ≤90 msec, (8), PASP/VTI*_LVOT_*>2 mmHg/cm [9,10].

For ventriculoarterial coupling, values were compared to the mean value of 1.1, found in healthy adults above 60 years of age, according to previous reports [11] and our unpublished data.

**RESULTS**

All the patients were admitted in the ICU after they had been intubated in the general ward. The majority of the patients, apart from 12, were immediately admitted after intubation. These 12 patients had stayed intubated in the general ward for a mean of 31±6 hours. For the patients that were transferred from another hospital, the time delay from intubation to ICU admission was 6 hours at most. Eighteen patients were excluded due to severe pulmonary embolism (PE); in 15, PE was confirmed with computed tomography Pulmonary Angiography (CTPA) (CTPA performed before ICU admission in 109), while three more patients were admitted due to acute severe shock and signs of Acute Core Pulmonale (ACP) [RV End Diastolic Area/ Left Ventricular End Diastolic Area (RVEDA/LVEDA)>0.6 with paradoxical septal motion], were considered to suffer from massive pulmonary embolism and received thrombolysis.

All measurements were obtained with transthoracic echocardiography. As it is presented in Table 2 LV volumes were not evaluated with 3D echocardiography in 71 patients. From these, 50 patients were evaluated with a machine not having the ability (program) to perform 3D measurements. In the rest 21 patients, LV volumes could not be obtained from the measurements, as the image was not clear enough and after consensus with all the doctors, we decided not to include these measurements. Concerning the RV volumes, they were not obtained in 84 patients. Removing the 50 patients with no 3D data, RV volumes could not be obtained in 34 patients (due to poor image quality) (feasibility of 3D echocardiography 27%). Among the 17 patients presenting with AF at the time of the echocardiographic study, the myocardial strain could not be evaluated in 11 due to technical issues (no strain imaging availability), while in all the rest 6, the myocardial strain was measured. Regarding 3D volume measurements, this could not be measured in 4/6.

The LV Ejection Fraction values assessed using 2D and 3D echocardiography were highly correlated (r 0.713, p<0.0001).

***RV function***

As already mentioned, RV-LS>-20% was present in almost the whole cohort 131/145 (90.3%) patients measured, while RV-LS≥-17 (average value reported in severe Covid-19 patients) was present in 90/145 (62%). From the rest of the variables indicating RV systolic dysfunction, TAPSE≤16 mm was present in 34/164 (20.7%) and RV Fractional Area Change, RVFAC ≤35% in 81/176 (46%), while RVEF≤44% (using 3D echocardiography) was present in 47/92 (51.1%) of patients.

From 164 patients with combined RVFAC and TAPSE measurements, 26 presented decreased both the RVFAC and TAPSE; fifty five patients with RVFAC≤35% had TAPSE >16 mm, while only 8 patients presented TAPSE ≤ 16 mm with normal RVFAC. In 27/36 (75%) of the patients having a mid-systolic notch, there were at least two more indices indicating increased. PASP/VTI*_LVOT_* and PASP/VTI*_RVOT_*>2 was present in 44/128 (32%) and 71/135 (52.6%) patients, using each estimation, respectively. PASP/VTI*_LVOT_* and PASP/VTI*_RVOT_* (initial and re-evaluation) were highly correlated (r 0.905 and r:0.855 respectively, both , p<0.0001). However, in our analysis as we have mentioned, we used the PASP/VTI*_LVOT_* value to indicate PVRs as VTI_LVOT_ is usually used to estimate the cardiac output and considered more accurate and repeatable than VTI*_RVOT._* PASP/VTI*_RVOT_* correlated with TAPSE: -0.340, p<0.0001 and RV-LS: 0.211, p=0.018.

***LV function***

Mean EF was nearly normal (57.8±1.1%); LV systolic dysfunction (LVEF<40%) was present in 21/176 (11.9%), while severely decreased (EF<30%) in 7 patients (4%). None of these patients had any known history of LV cardiomyopathy. However, LV-LS was reduced (-13.3±0.3%), 127/145 (87.5%) patients presented LVLS >-18%, while in 82/145 (56.5%) LVLS was >-15.9% (Lower Limit Normal) and >-14 in 67 (46.2%) (Table 2). Twenty-three patients (12.5%) had a history of Coronary Artery Disease (CAD). In five of them, EF before ICU admission was <40% and were excluded (Figure 1). In the rest 18, EF was preserved (>40%) according to their personal records. Among the 21 patients presenting LVEF<40% upon ICU admission, four patients (19%) had previously known CAD.

**Pericardial effusion**

Two patients presented moderate effusions (1.3-2 cm). The first patient presented signs of right atrial compression during the course of a septic episode, occurring during the fifth ICU day, which was managed with fluid loading. The second underwent pericardiocentesis during the tenth ICU day. A third patient, who was excluded from the analysis (Figure), presented moderate pericardial effusion which was drained upon ICU admission as he presented signs of cardiogenic shock. The pericardial fluid (in both) was hemorrhagic with a lymphocyte predominance, while SARS-COV-2 was not detected (PCR negative).

**Echocardiographic Time course of COVID-19 cardiac involvement**

Upon re-evaluation C*_RS_* correlated to respiratory function (PaO_2_/FiO_2_, r-0.645, p<0.0001; PaCO_2_, r:-0.735, p<0.0001), RV function (RV s’, r:-0.478 p<0.0001; RV-LS, r:0.345 p=0.001) and RV afterload (PASP/VTI*_RVOT_*, r: 0.535, p<0.0001, PASP, r:0.570 p<0.0001) but not to RVEDA/LVEDA, r:0.188 p=0.087.

The cardiac function upon re-evaluation did not differ between patients with increased and normal troponin levels (Supplementary Table 8).

**REFERENCES**

1. Rudski LG, Lai WW, Afilalo J, et al. Guidelines for the echocardiographic assessment of the right heart in adults: a report from the American Society of Echocardiography endorsed by the European Association of Echocardiography, a registered branch of the European Society of Cardiology, and the Canadian Society of Echocardiography. J Am Soc Echocardiogr. 2010;23(7):685-713; quiz 786-8. doi: 10.1016/j.echo.2010.05.010
2. Lang RM, Badano LP, Belohlavek M, et al. Current and evolving echocardiographic techniques for the quantitative evaluation of cardiac mechanics: ASE/EAE consensus statement on methodology and indications endorsed by the Japanese Society of Echocardiography. Eur J Echocardiogr. 2011;12(3):167-205. doi: 10.1093/ejechocard/jer021.
3. Takahama H, McCully RB, Frantz RP, et al. Unraveling the RV Ejection Doppler Envelope: Insight Into Pulmonary Artery Hemodynamics and Disease Severity. JACC Cardiovasc Imaging. 2017;10(10 Pt B):1268-1277. doi: 10.1016/j.jcmg.2016.12.021.
4. Tello K, Wan J, Dalmer A, et al. Validation of the Tricuspid Annular Plane Systolic Excursion/Systolic Pulmonary Artery Pressure Ratio for the Assessment of Right Ventricular-Arterial Coupling in Severe Pulmonary Hypertension. Circ Cardiovasc Imaging. 2019;12(9):e009047. doi: 10.1161/CIRCIMAGING.119.009047. Epub 2019 Sep 10.
5. Abbas AE, Fortuin FD, Schiller NB, et al. A simple method for noninvasive estimation of pulmonary vascular resistance. J Am Coll Cardiol. 2003;41(6):1021-7. doi: 10.1016/s0735-1097(02)02973-x.
6. Roule V, Labombarda F, Pellissier A, et al. Echocardiographic assessment of pulmonary vascular resistance in pulmonary arterial hypertension. Cardiovasc Ultrasound. 2010 Jun 7;8:21. doi: 10.1186/1476-7120-8-21.
7. Lang RM, Bierig M, Devereux RB, et al. Chamber Quantification Writing Group; American Society of Echocardiography's Guidelines and Standards Committee; European Association of Echocardiography. Recommendations for chamber quantification: a report from the American Society of Echocardiography's Guidelines and Standards Committee and the Chamber Quantification Writing Group, developed in conjunction with the European Association of Echocardiography, a branch of the European Society of Cardiology. J Am Soc Echocardiogr. 2005;18(12):1440-63. doi: 10.1016/j.echo.2005.10.005.
8. Opotowsky AR, Clair M, Afilalo J, et al. A simple echocardiographic method to estimate pulmonary vascular resistance. Am J Cardiol. 2013;112(6):873-82. doi: 10.1016/j.amjcard.2013.05.016.
9. Ferrara F, Rudski LG, Vriz O, et al. Physiologic correlates of tricuspid annular plane systolic excursion in 1168 healthy subjects. Int J Cardiol. 2016 Nov 15;223:736-743. doi: 10.1016/j.ijcard.2016.08.275. Epub 2016 Aug 18. PMID: 27573598.

**Table S1**

**Demographics in the whole cohort**

| **Whole cohort (n=176)** | |
| --- | --- |
| **DEMOGRAPHICS AND ANTHROPOMETRICS** | |
| **Age, years** | 67.2± 0.8 |
| **Sex (male), n, %** | 121 (65.8) |
| **BMI, kg/m^2^** | 27.4± 0.4 |
| **PREVIOUS MEDICAL HISTORY, N (%)** | |
| **Arterial hypertension** | 112 (63.6) |
| **Diabetes Mellitus** | 51 (29) |
| **Coronary Artery Disease** | 18 (10) |
| **Dyslipidaemia** | 54 (30.7) |
| **Cerebrovascular disease** | 29 (16.5) |
| **COPD** | 17 (9.7) |
| **Malignancy** | 12 (6.8) |
| **Smoking Habitus** | 47 (26.7) |
| **DISEASE SEVERITY AND LABORATORY DATA** | |
| **Infection day on ICU admission** | 11.2± 0.44 |
| **APACHE II score** | 16.86 ± 0.55 |
| **SOFA score** | 7.86± 0.16 |
| **WBC, x 10^6^/L (4,000-10,000)** | 11,440.25± 551.36 |
| **Lymphocytes, x 10^6^/L (1,000-4,800)** | 703.64± 51.36 |
| **D dimers, ng/ml (<300)** | 1525.99± 153.26 |
| **CRP, mg/dl (<0.5)** | 8.71± 0.72 |
| **Ferritin, ng/ml (24-336)** | 2160.56± 576.9 |
| **Urea, mg/dl (6-50)** | 67.19± 3.95 |
| **Creatinine, mg/dl (0.7-1.3)** | 1.08± 0.07 |
| **Urea/creatinine** | 65.04± 2.65 |
| **Troponin, ng/ml (0-0.04)** | 0.34± 0.07 |
| **Troponin (mean values in the patients with troponin >0.04 ng/ml)** | 0.67±0.13 (n=86) |
| **Troponin (mean values in the patients with troponin ≤0.04 ng/ml)** | 0.028±0.001 (n=90) |
| Data are expressed as mean ± standard error of means.  APACHE II, Acute Physiology and Chronic Health Evaluation; BMI, Body Mass Index; COPD, Chronic Obstructive Pulmonary Disease; CRP, C-Reactive Protein, ICU, Intensive Care Unit; PEEP, Positive End Expiratory Pressure; SOFA, Sequential Organ Failure Assessment; WBC, White Blood Cells; | |

**Table S2.**

**Demographic data and respiratory variables in Covid-19 ARDS patients according to the presence of increased troponin levels.**

|  | **TNI≤0.04 ng/ml**  **(n=90)** | **TNI > 0.04 ng/ml (n=86)** |  |
| --- | --- | --- | --- |
|  | **Admission** | **Day 10** | **P-value** |
| **Age** | 66.9± 1.1 (90) | 67.6 ± 1.1 (86) | 0.511 |
| **Sex (%)** | 60 (67) | 54 (63) | 0.378 |
| **BMI, kg/m^2^** | 27.6 ± 0.5 (90) | 27.3 ± 0.6 (86) | 0.642 |
| **Infection day on ICU admission** | 10.5± 0.6 | 11.8± 0.6 | 0.093 |
| **APACHE II score** | 16.8 ± 0.7 | 17.1 ± 0.9 | 0.812 |
| **SOFA score** | 7.8± 0.2 | 7.9± 0.3 | 0.782 |
| **WBC, x 10^6^/L (4,000-10,000)** | 11,679.5± 867.8 | 11,066.4± 707.9 | 0.483 |
| **Lymphocytes, x 10^6^/L (1,000-4,800)** | 800.3± 89.8 | 596.7± 40 | 0.047 |
| **D dimers, ng/ml (<300)** | 1494.5± 210.4 | 1418.8± 180.4 | 0.825 |
| **CRP, mg/dl (<0.5)** | 9.7± 1 | 8± 1 | 0.215 |
| **Ferritin, ng/ml (24-336)** | 2296.7± 988.8 | 2066.5± 569.2 | 0.826 |
| **Urea, mg/dl (6-50)** | 59.9± 4.2 | 66.4±4.9 | 0.371 |
| **Creatinine, mg/dl (0.7-1.3)** | 0.98± 0.07 | 1.2± 0.1 | 0.227 |
| **Urea/creatinine** | 67.2± 3.9 | 62.6± 3.4 | 0.390 |
| **Troponin, ng/ml** | 0.03± 0.001 | 0.67± 0.1 | <0.0001 |
| **Respiratory variables** | | | |
| **Vt, ml/kg** | 6.8 ± 0.1 | 7 ± 0.2 | 0.233 |
| **PEEP, cm H_2_O** | 11.7 ± 0.3 | 11.4 ± 0.3 | 0.475 |
| **PaO_2_/FiO_2_, mm Hg** | 90.6 ±4.4 | 95 ±5.6 | 0.545 |
| **PaCO_2_, mm Hg** | 54.5 ± 2.4 | 52.8 ± 2.1 | 0.595 |
| **C*_RS_*, ml/cm H_2_O** | 34.9 ± 1.3 | 32.4 ± 1.4 | 0.177 |
| **P*_plat_*, cmH_2_O** | 25.6 ± 0.5 | 26.5± 0.5 | 0.211 |
| **DP, cmH_2_O** | 13.6±0.4 | 15.1±0.6 | 0.026 |
| **Outcomes** | | | |
| **Sepsis, n (%)** | 50 (55.5) | 48 (55.8) | 0.548 |
| **10-day survival, n (%)** | 77 (75.6) | 62 (72) | 0.05 |
| **ICU Survival, n (%)** | 38 (42.2) | 26 (30) | 0.136 |
| Data are expressed as mean ± standard error of means;  APACHE II, Acute Physiology and Chronic Health Evaluation; ARDS, Acute Respiratory Distress Syndrome; BMI, Body Mass Index; CRP, C-Reactive Protein; C*_RS_*, Respiratory System Compliance; DP, Driving Pressure; ICU, Intensive Care Unit; PaCO2, Partial Carbon Dioxide Pressure; PaO2/FiO2, Partial Oxygen Pressure/Fraction of Inspired Oxygen; PEEP, Positive End Expiratory Pressure; Pplat, Plateau Pressure; SOFA; Sequential Organ Failure Assessment; Vt, Tidal Volume; WBC, White Blood Cells. | | | |

**Table S3. Echocardiogaphic Variables in the whole cohort upon ICU admission and upon the 10^th^ ICU day**

| **Echocardiographic Variable** | **Covid-19 ARDS patients (n)*^a^*** | | **p-value** |
| --- | --- | --- | --- |
|  | **ICU admission (n=176)** | **Follow up (n=127)** |  |
| **Demographics** | | | |
| **Age, years** | 65.9± 1.5 | 67.4 ± 1.0 | 0.267 |
| **Sex, %** | 39 (69.6%) | 80 (66.7%) | 0.725 |
| **APACHE II score** | 16.2 ± 0.9 | 17.3 ± 0.7 | 0.348 |
| **SOFA score** | 7.5± 0.2 | 8.05± 0.2 | 0.102 |
| **Echocardiographic variables: Left Ventricle** | | | |
| LVEDD, mm | 4.59 ± 0.06 (176) | 4.62 ± 0.08 (127) | 0.789 |
| IVS, mm | 0.92 ± 0.01 (176) | 0.9 ± 0.01 (127) | 0.956 |
| VTI*_LVOT_*, cm | 20.2± 0.4 (169) | 20.8± 0.3 (127) | 0.274 |
| LVEDA, cm2 | 28.2± 0.5 (176) | 27.6± 0.5 (127) | 0.409 |
| LVESA, cm2 | 14.2± 0.8 (176) | 14.1± 0.6 (127) | 0.058 |
| LVEDV, ml (2D) | 86.6 ± 2.4 (176) | 83.2 ± 2.2 (127) | 0.347 |
| LVESV, ml (2D) | 37 ± 1.9 (176) | 32.7 ± 1.5 (127) | 0.137 |
| EF, % | 59.1 ± 1.2 (176) | 60.5 ± 1.4 (127) | 0.476 |
| SV, ml (Simpson’s) | 48.1± 1.8 (176) | 50.1± 1.6 (127) | 0.101 |
| LVEDV, ml (3D) | 83.2± 2.2 (105) | 84.9± 2.1 (72) | 0.599 |
| LVESV, ml (3D) | 35.3 ± 1.5 (105) | 35 ± 1.4 (72) | 0.860 |
| EF (3D), % | 57.8±1.1 (105) | 58.5±1.1 (72) | 0.661 |
| LV-LS, % | -13.3 ± 0.3 (145) | -14.2 ± 0.5 (99) | 0.885 |
| LV-LS >-18% | 127/145 (87.5%) | 77/99 (77.7%) | 0.039 |
| LV S’, cm/s | 10.4 ± 0.5 (149) | 10.5 ± 0.5 (96) | 0.885 |
| **Echocardiographic variables: Right Ventricle** | | | |
| RVEDA/LVEDA | 0.82± 0.02 (176) | 0.8± 0.03 (127) | 0.689 |
| RVEDA, cm^2^ | 22.2 ± 0.4 (176) | 21.3 ± 0.6 (127) | 0.219 |
| RVESA, cm^2^ | 13.9 ± 0.3 (176) | 13.1 ± 0.5 (127) | 0.194 |
| RV FAC, % | 36.6 ± 0.9 (176) | 39.1 ± 0.1 (127) | 0.048 |
| RVEDV*^b^*, ml (3D) | 106.9 ± 4 (92) | 112.7 ± 4.5 (73) | 0.339 |
| RVESV*^b^*, ml (3D) | 64.1 ± 3 (92) | 66.3 ± 3 (73) | 0.618 |
| RVEF*^b^* (%) (3D) | 41.1 ± 1.3 (92) | 41.1 ± 1.3 (73) | 0.995 |
|  |  |  |  |
| TAPSE, mm | 20.4±0.4 (164) | 21.9±0.1 (124) | 0.159 |
| RV S’, cm/sec | 16.1 ± 0.44 (149) | 16.4 ± 0.43 (127) | 0.637 |
| RV IVA, m/sec^2^ | 3.78± 0.1 (149) | 3.95 ± 0.1 (127) | 0.231 |
| RV-LS, % | -14.4 ± 0.4 (140) | -14.6 ± 0.5 (106) | 0.895 |
| VTI*_RVOT,_* cm | 16.2 ± 0.3 (142) | 17.5 ± 0.4 (101) | 0.014 |
| PASP, mmHg | 33 ± 1.1 (150) | 30.1 ± 1.3 (109) | 0.085 |
| PASP/VTI*_LVOT_*, mmHg/cm | 1.9 ± 0.1 (128) | 1.5 ± 0.1 (108) | 0.035 |
| PASP/VTI*_RVOT_*, mmHg/cm | 2.29 ± 0.1 (135) | 1.9 ± 0.1 (96) | <0.0001 |
| Pulmonary AcT, msec | 65.3 ± 1.5 (142) | 72 ± 2.1 (101) | 0.035 |
| RVOT notch | 36 (25.4%) (142) | 17 (16.8%) (101) | 0.157 |
| VAC_R,_ mm/mmHg | 0.80 ± 0.06 (144) | 0.95 ± 0.09 (101) | 0.142 |
| IVC*, cm | 2.29 ± 0.04 (153) | 2 ± 0.06 (109) | <0.0001 |
| ΔIVC, % | 11.7±2.6 (153) | 19±2.3 (109)^ | 0.04 |
|  |  |  |  |
| *Maximum diameter measured during inspiration under Mechanical Ventilation.  ^Seventeen patients were on a pressure support mode  Data are expressed as mean ± standard error of means; *^a^* numbers in parenthesis refer to the actual number of patients that the variable was measured, ^b^ 3D measurements.  AcT, Acceleration Time; APACHE II, Acute Physiology and Chronic Health Evaluation II score; EF: Ejection Fraction; ΔIVC: respiratory variability in Inferior Vena Cava diameter [ (IVCmax-IVCmin)/IVCmin]; LV-LS, longitudinal strain of the left ventricle; IVA, Isovolumic Acceleration; IVC, Inferior Vena Cava; IVS, Interventricular septum; LVEDA, Left Ventricular End Diastolic Area; LVESA, Left Ventricular End Systolic Area; LVEDV, Left Ventricular End Diastolic Volume; LVESV, Left Ventricular End Systolic Volume; LV S’, Systolic tissue doppler velocity measured at the lateral mitral annulus; PASP, Pulmonary Artery Systolic Pressure; PASP/VTI*_LVOT_*, Pulmonary Artery Systolic Pressure to Left Ventricular Outflow Tract Velocity Time Integral ratio; RVEDA/LVEDA, Right Ventricular End Diastolic Area to Left Ventricular End Diastolic Area; RVEDV, Right Ventricular End Diastolic Volume; RVEF, Right Ventricular Ejection Fraction; RVESV, Right Ventricular End Systolic Volume; RVFAC, Right Ventricular Fractional Area Change; RV-IVA, Right Ventricular Isovolumic Acceleration; RV-LS, right ventricular free wall longitudinal strain; RV S’, Systolic tissue doppler velocity measured at the lateral tricuspid annulus; SOFA score, Sequential Organ Failure Assessment Score; SV: Stroke Volume; TAPSE: Tricuspid Annular Plane Systolic Excursion; VAC*_R_*, Right Ventricular to Pulmonary artery coupling; VTI*_LVOT_*, Left Ventricular Outflow Tract Velocity Time Integral; VTI*_RVOT,_*  Right Ventricular Outflow Tract Velocity Time Integral; | | | |

**Table S4. Clinical data and Echocardiogaphic Variables of initial evaluation between 10-day survivors and non-survivors**

| **Echocardiographic Variable** | **Covid-19 ARDS patients (n)*^a^*** | | **p-value** |
| --- | --- | --- | --- |
|  | **10-day survivors (n=137)** | **10-day non-survivors (n=39)** |  |
| Age | 67.7 ± 0.06 | 66.1± 0.06 | 0.411 |
| BMI | 27.4± 0.5 | 27.5 ± 0.6 | 0.908 |
| APACHE II | 17± 0.6 | 16.9 ± 1.5 | 0.922 |
| SOFA score | 7.9± 0.2 | 8 ± 0.4 | 0.791 |
| PaO2/FiO2 | 89.5 ± 3.8 | 106.3± 9.2 | 0.062 |
| PaCO2 | 53.8 ± 2.1 | 52.6± 2.2 | 0.724 |
| CRS | 34.7 ± 1.1 | 29.6± 1.7 | 0.024 |
| Pplat | 25.6 ± 0.4 | 28± 0.8 | 0.008 |
| DP | 14 ± 0.4 | 15.4± 0.8 | 0.097 |
| Noradrenaline | 0.45 ± 0.06 | 0.73± 0.3 | 0.113 |
| ScVO2 | 69.4 ± 1.2 | 67.9± 1.8 | 0.519 |
| D-Dimers | 1555 ± 153 | 894.3± 243 | 0.079 |
| Ferritin | 1538.8 ± 256.2 | 4980.5± 2915.2 | 0.021 |
| Troponin | 0.21± 0.06 | 0.78 ± 0.19 | <0.0001 |
| **Echocardiographic variables: Left Ventricle** | | | |
| LVEDD, mm | 4.6 ± 0.06 | 4.5 ± 0.07 | 0.789 |
| VTI*_LVOT_*, cm | 20.3± 0.4 | 19.9± 0.9 | 0.745 |
| LVEDA, cm2 | 28.3± 0.5 | 28.1± 1.04 | 0.855 |
| LVESA, cm2 | 15.4± 0.8 | 18.8± 2 | 0.074 |
| LVEDV, ml (2D) | 85.9 ± 2.8 | 88.9 ± 4.9 | 0.605 |
| LVESV, ml (2D) | 34.8± 2 | 44.5 ± 4.9 | 0.036 |
| EF, % | 60.9 ± 1.2 | 53.4 ± 2.8 | 0.006 |
| SV, ml (Simpson’s) | 50.1± 2 | 39.3± 3.9 | 0.018 |
| LVEDV, ml (3D) | 82.6± 2.4 | 84.9± 5.9 | 0.687 |
| LVESV, ml (3D) | 34.3 ± 1.3 | 40.4 ± 6 | 0.124 |
| EF (3D), % | 58.4±1.1 | 54.8±3.9 | 0.236 |
| LV-LS, % | -13.4 ± 0.4 | -13.3 ± 0.7 | 0.913 |
| **Echocardiographic variables: Right Ventricle** | | | |
| RVEDA/LVEDA | 0.82± 0.02 | 0.77± 0.05 | 0.313 |
| ACP, n (%) | 26 (19%) | 9 (23.1%) | 0.532 |
| RVEDA, cm^2^ | 22.5 ± 0.5 | 20.9 ± 0.9 | 0.137 |
| RVESA, cm^2^ | 13.9 ± 0.3 | 14 ± 0.7 | 0.902 |
| RV FAC, % | 37.7 ± 1 | 31.7 ± 2 | 0.011 |
| RVEDV*^b^*, ml (3D) | 116 ± 5.4 | 89.8 ± 3.4 | 0.001 |
| RVESV*^b^*, ml (3D) | 68.5 ± 4 | 55.9 ± 3.5 | 0.047 |
| RVEF*^b^* (%) (3D) | 42.4 ± 1.5 | 38.7 ± 2.5 | 0.191 |
| RV SV | 48.6 ± 2.4 | 33.9 ± 2.4 | <0.0001 |
| TAPSE, mm | 20.6±0.4 | 20±0.8 | 0.470 |
| RV S’, cm/sec | 16.2 ± 0.5 | 15.9 ± 1 | 0.737 |
| RV IVA | 3.8± 0.1 | 3.6 ± 0.2 | 0.270 |
| RV-LS, % | -14.5 ± 0.5 | -14.5 ± 0.7 | 0.981 |
| VTI*_RVOT,_* cm | 16.7 ± 0.4 | 14.7 ± 0.6 | 0.011 |
| PASP, mmHg | 33.1 ± 1.3 | 32.6 ± 2.1 | 0.861 |
| PASP/VTI*_LVOT_*, mmHg/cm | 1.8 ± 0.1 | 2 ± 0.3 | 0.497 |
| Pulmonary AcT, msec | 65.1 ± 1.7 | 65.6 ± 2.9 | 0.879 |
| RVOT notch | 26 (19%) | 10 (25.6) | 0.211 |
| VAC_R,_ mm/mmHg | 0.84 ± 0.08 | 0.67 ± 0.05 | 0.228 |
| IVC*, cm | 2.31 ± 0.04 | 2.2 ± 0.07 | 0.517 |
| ΔIVC, % | 12.8±3.4 | 7.8±1.3 | 0.431 |
| **Echocardiographic variables: Pericardium** | | | |
| **Pericardial effusion** | 48/137 (35%) | 22/39 (56.4%) | 0.015 |
| *Maximum diameter measured during inspiration under Mechanical Ventilation.  ^Seventeen patients were on a pressure support mode  Data are expressed as mean ± standard error of means; *^a^* numbers in parenthesis refer to the actual number of patients that the variable was measured, ^b^ 3D measurements.  ACP, Acute Cor Pulmonale; AcT, Acceleration Time; APACHE II, Acute Physiology and Chronic Health Evaluation II score; ARDS, Acute Respiratory Distress Syndrome; BMI, Body Mass Index; CRP, C-Reactive Protein; C*_RS_*, Respiratory System Compliance; DP, Driving Pressure; EF: Ejection Fraction; ΔIVC: respiratory variability in Inferior Vena Cava diameter [ (IVCmax-IVCmin)/IVCmin]; ICU, Intensive Care Unit; PaCO2, IVC, Inferior Vena Cava; LV-LS, longitudinal strain of the left ventricle; LVEDA, Left Ventricular End Diastolic Area; LVESA, Left Ventricular End Systolic Area; LVEDV, Left Ventricular End Diastolic Volume; LVESV, Left Ventricular End Systolic Volume; LV S’, Systolic tissue doppler velocity measured at the lateral mitral annulus; PaCO2, Partial Carbon Dioxide Pressure; PaO2/FiO2, Partial Oxygen Pressure/Fraction of Inspired Oxygen; PEEP, Positive End Expiratory Pressure; Pplat, Plateau Pressure; PASP, Pulmonary Artery Systolic Pressure; PASP/VTI*_LVOT_*, Pulmonary Artery Systolic Pressure to Left Ventricular Outflow Tract Velocity Time Integral ratio; RVEDA/LVEDA, Right Ventricular End Diastolic Area to Left Ventricular End Diastolic Area; RVEDV, Right Ventricular End Diastolic Volume; RVEF, Right Ventricular Ejection Fraction; RVESV, Right Ventricular End Systolic Volume; RVFAC, Right Ventricular Fractional Area Change; RV-IVA, Right Ventricular Isovolumic Acceleration; RV-LS, right ventricular free wall longitudinal strain; RV S’, Systolic tissue doppler velocity measured at the lateral tricuspid annulus; SOFA score, Sequential Organ Failure Assessment Score; SV: Stroke Volume; TAPSE: Tricuspid Annular Plane Systolic Excursion; VAC*_R_*, Right Ventricular to Pulmonary artery coupling; Vt, Tidal Volume; VTI*_LVOT_*, Left Ventricular Outflow Tract Velocity Time Integral; VTI*_RVOT,_*  Right Ventricular Outflow Tract Velocity Time Integral WBC, White Blood Cells. | | | |

**Table S5. Baseline Characteristics and outcome between survivors and non-survivors.**

| **Echocardiographic Variable** | **Covid-19 ARDS survivors (n=56)** | **Covid-19 ARDS non-survivors (n=120)** | **p-value** |
| --- | --- | --- | --- |
| **Baseline characteristics** | | | |
| **Age, years** | 65.9± 1.5 | 67.4 ± 1.0 | 0.267 |
| **Sex, %** | 39 (69.6%) | 80 (66.7%) | 0.725 |
| **BMI, kg/m^2^** | 27.4 ± 0.6 | 27.4 ± 0.5 | 0.976 |
| **Infection day on ICU admission** | 11.2± 0.7 | 11.1± 0.6 | 0.958 |
| **APACHE II score** | 16.2 ± 0.9 | 17.3 ± 0.7 | 0.348 |
| **SOFA score** | 7.5± 0.2 | 8.05± 0.2 | 0.102 |
| **WBC, x 10^6^/L (4,000-10,000)** | 11,132.1± 821.7 | 11,607.8± 726.8 | 0.682 |
| **Lymphocytes, x 10^6^/L (1,000-4,800)** | 696.6± 87.3 | 707.5± 63.8 | 0.920 |
| **D dimers, ng/ml (<300)** | 1981.8± 326.5 | 1268.3± 148.2 | 0.025 |
| **CRP, mg/dl (<0.5)** | 9.5± 1.4 | 8.3± 0.8 | 0.438 |
| **Ferritin, ng/ml (24-336)** | 1250.4± 173.1 | 2746.6± 937.9 | 0.207 |
| **Urea, mg/dl (6-50)** | 72.4± 6.4 | 58.1± 3.3 | 0.03 |
| **Creatinine, mg/dl (0.7-1.3)** | 1.13± 0.1 | 1.05± 0.09 | 0.570 |
| **Urea/creatinine** | 63.9± 3.8 | 65.7± 3.6 | 0.747 |
| **Troponin, ng/ml** | 0.05± 0.01 | 0.49± 0.1 | 0.001 |
| **Respiratory variables** | | | |
| **Vt, ml** | 6.8 ± 0.1 | 6.9 ± 0.08 | 0.983 |
| **PEEP, cm H_2_O** | 11.7 ± 0.4 | 11.5 ± 0.3 | 0.978 |
| **PaO_2_/FiO_2_, mm Hg** | 86.3 ±5.2 | 95.9 ±4.7 | 0.196 |
| **PaCO_2_, mm Hg** | 48.9 ± 1.9 | 55.4 ± 1.9 | 0.061 |
| **C*_RS_*, ml/cm H_2_O** | 38.7 ± 1.5 | 31 ± 1.1 | <0.0001 |
| **P*_plat_*, cmH_2_O** | 23.9 ± 0.5 | 27.3± 0.4 | <0.0001 |
| **DP, cmH_2_O** | 12.3±0.4 | 15.4±0.5 | <0.0001 |
| **Outcomes** | | | |
| **Sepsis, n (%)** | 25/56 (40.6) | 72/120 (60) | 0.034 |
| **Sepsis until the 10^th^ ICU day, n (%)** | 3 (5.3) | 37 (30.8) | 0.773 |
| **ICU Survival, days** | 26±3.6 | 13.2±0.8 | <0.0001 |
| Data are expressed as mean ± standard error of means;  APACHE II, Acute Physiology and Chronic Health Evaluation; ARDS, Acute Respiratory Distress Syndrome; BMI, Body Mass Index; CRP, C-Reactive Protein; C*_RS_*, Respiratory System Compliance, DP, Driving Pressure; ICU, Intensive Care Unit; PaCO2, Partial Carbon Dioxide Pressure; PaO2/FiO2, Partial Oxygen Pressure/Fraction of Inspired Oxygen; PEEP, Positive End Expiratory Pressure; Pplat, Plateau Pressure; SOFA; Sequential Organ Failure Assessment; Vt, Tidal Volume; WBC, White Blood Cells; | | | |

**Table S6. Univariate and Multivariate Regression Models to identify predictors of survival.**

| **MODEL 1** | | | | | | |
| --- | --- | --- | --- | --- | --- | --- |
|  | **Univariate Analysis** | | | **Multivariate Analysis** | | |
|  | OR | 95% CI | P-value | OR | 95% CI | P-value |
| d-dimers | 1.000 | 1.000-1.000 | 0.032 |  |  |  |
| Urea | 0.990 | 0.981-0.999 | 0.035 |  |  |  |
| Troponin | 66.549 | 2.018-2195 | 0.019 |  |  |  |
| C*_RS_* | 0.945 | 0.916-0.975 | <0.001 | 0.842 | 0.721-0.982 | 0.028 |
| Pplateau | 1.170 | 1.078-1.270 | <0.0001 | 1.425 | 1.024-1.982 | 0.036 |
| Driving Pressure | 1.236 | 1.111-1.375 | <0.0001 |  |  |  |
| RVEDV | 0.984 | 0.971-0.998 | 0.022 |  |  |  |
| RVESV | 0.974 | 0.954-0.994 | 0.013 |  |  |  |
| Pericardial effusion | 2.076 | 1.053-4.096 | 0.035 |  |  |  |
| **MODEL 2** | | | | | | |
|  | **Univariate Analysis** | | | **Multivariate Analysis** | | |
| LVOT VTI | 0.901 | 0.817-0.992 | 0.035 |  |  |  |
| LV-LS | 1.397 | 1.217-1.604 | <0.0001 | 1.881 | 1.105-3.203 | 0.020 |
|  |  |  |  |  |  |  |
| RVEDA/LVEDA | 61.103 | 6.629-563.190 | <0.0001 |  |  |  |
| RVEDA | 1.185 | 1.099-1.277 | <0.0001 |  |  |  |
| RVESA | 1.231 | 1.109-1.367 | <0.0001 |  |  |  |
| RVEDV | 1.015 | 1.001-1.029 | 0.040 |  |  |  |
| RVESV | 1.033 | 1.009-1.057 | 0.007 |  |  |  |
| RVEF | 0.004 | 0.000-0.756 | 0.039 |  |  |  |
| RV S’ | 0.000 | 0.000-0.000 | <0.0001 |  |  |  |
| RV-LS | 1.240 | 1.125-1.368 | <0.0001 |  |  |  |
| RVOT VTI | 0.753 | 0.657-0.862 | <0.0001 |  |  |  |
| PASP | 1.166 | 1.100-1.235 | <0.0001 |  |  |  |
| PASP/VTI*_RVOT_* | 14.458 | 5.148-40.608 | <0.0001 |  |  |  |
| PAcT | 0.949 | 0.925-0.974 | <0.0001 |  |  |  |
| RVOT notch | 2.234 | 0.670-7.456 | 0.191 |  |  |  |
| VAC_R_ | 0.000 | 0.000-0.000 | <0.0001 |  |  |  |
| **MODEL 3** | | | | | | |
|  | **Univariate Analysis** | | | **Multivariate Analysis** | | |
|  |  |  |  |  |  |  |
| ΔLV-LS | 0.584 | 0.334-1.024 | 0.060 |  |  |  |
| ΔRVEDA | 33.446 | 6.503-172.006 | <0.0001 |  |  |  |
| ΔRVESA | 25.225 | 5.067-125.565 | <0.0001 |  |  |  |
| ΔRV S’ | 0.166 | 0.053-0.519 | 0.002 |  |  |  |
| ΔRV-LS | 0.209 | 0.077-0.570 | 0.002 | 0.032 | 0.001-0.908 | 0.044 |
| ΔRVEDV | 38411.258 | 86.601-1,7037,059.04 | 0.001 |  |  |  |
| ΔRVESV | 312.663 | 10.171-9611.524 | 0.001 |  |  |  |
| ΔPASP/VTI*_RVOT_* | 27.325 | 5.858-237.468 | <0.0001 | 78.269 | 2.578-2376.234 | 0.012 |
| ΔRV FAC | 0.764 | 0.377-1.548 | 0.454 |  |  |  |
| ΔPAcT | 0.013 | 0.002-0.094 | <0.0001 |  |  |  |
| ΔRVEDA/LVEDA | 0.095 | 0.023-0.403 | 0.001 |  |  |  |
| AcT, Acceleration Time; C*_RS_*, static compliance of the respiratory system; DP, Driving Pressure; EF, Ejection Fraction; FiO_2_, fraction of inspired oxygen; LV, Left Ventricle; LVOT, Left Ventricular Outflow Tract; LV-LS, longitudinal strain of the left ventricle; PASP, Pulmonary Artery Systolic Pressure; PASP/VTI*_LVOT_*, Pulmonary Artery Systolic Pressure to Left Ventricular Outflow Tract Velocity Time Integral ratio; PASP/VTI*_RVOT_*, P*_pl,_* Pleural Pressure; RV, Right Ventricle; RVEDA/LVEDA, Right Ventricular End Diastolic Area to Left Ventricular End Diastolic Area; RVEDV, Right Ventricular End Diastolic Volume; RVESV, Right Ventricular End Systolic Volume; RVEF, Right Ventricular Ejection Fraction; RV-LS, right ventricular longitudinal strain; RV S’, systolic tissue doppler velocity measured at the lateral tricuspid annulus; TAPSE: Tricuspid Annular Plane Systolic Excursion; VAC*_R_*, ventriculoarterial coupling of the right ventricle to the pulmonary artery; VTI*_LVOT_*, Left Ventricular Outflow Tract Velocity Time Integral; | | | | | | |

Model 1. Regression model testing variables that significantly differed upon admission between survivors and non-survivors as independent predictors.

Model 2. Regression model testing variables that significantly differed upon re-evaluation between survivors and non-survivors as independent predictors.

Model 3. Regression model testing variables indicating the change in echocardiographic measurements that significantly differed between survivors and non-survivors as independent predictors.

**Table S7. Bland and Aldman scatter plots to estimate interobserver variability for different RV measurements.**


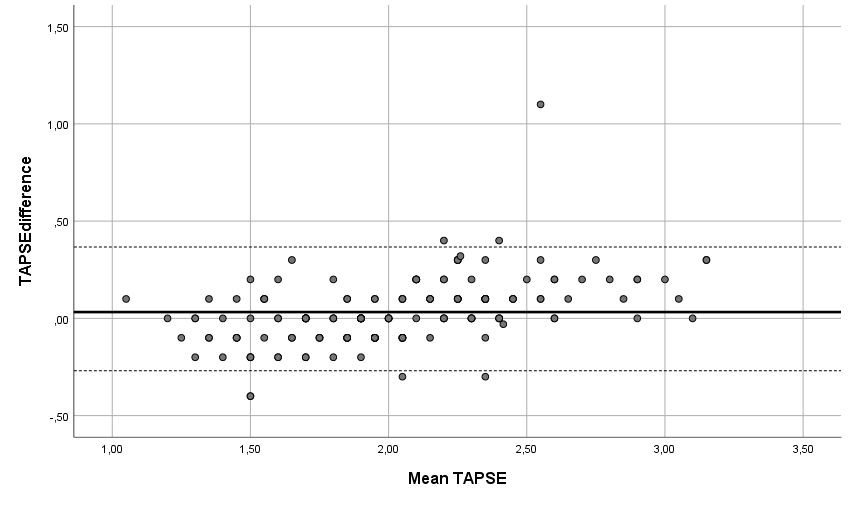


Bland & Altman scatter plot for the TAPSE score. Solid black line refers to the mean difference (0,0323), dashed line illustrates the upper and lower bound of the 95% confidence interval of the difference.


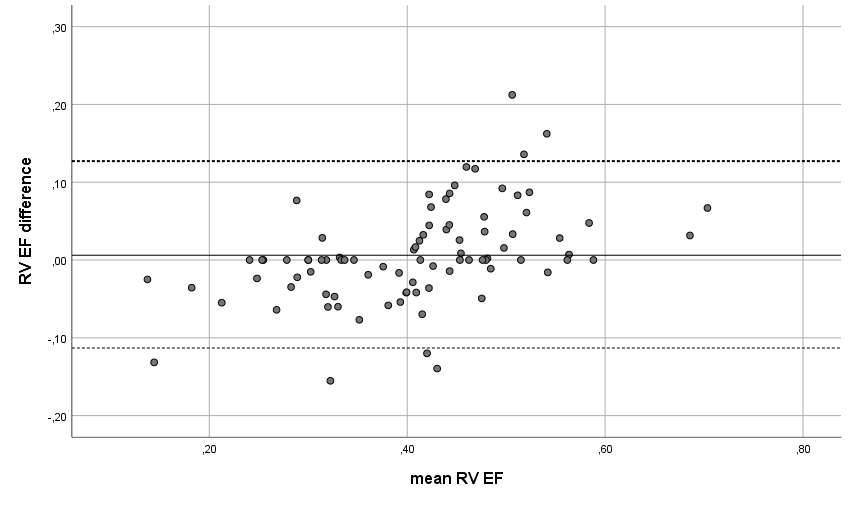


Bland & Altman scatter plot for RV EF. Solid black line refers to the mean difference (0.0061), dashed line illustrates the upper and lower bound of the 95% confidence interval of the difference.


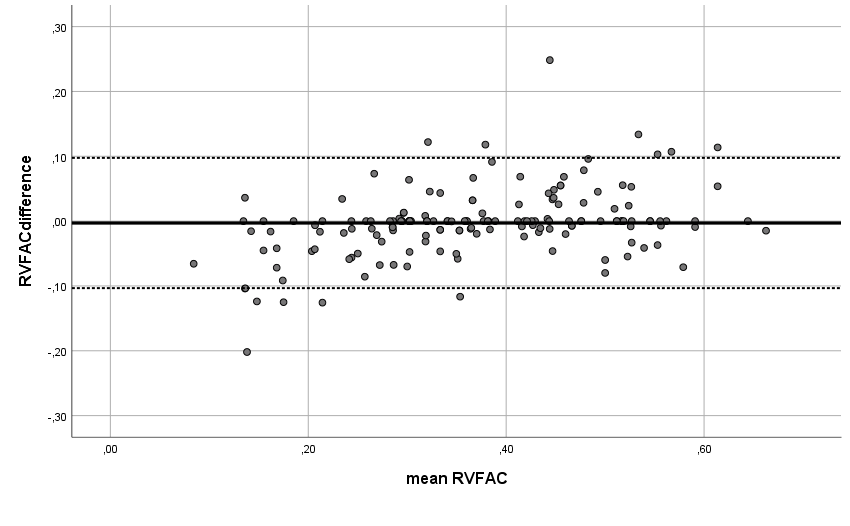


Bland & Altman scatter plot for the RV FAC. Solid black line refers to the mean difference (0.0003), dashed line illustrates the upper and lower bound of the 95% confidence interval of the difference.


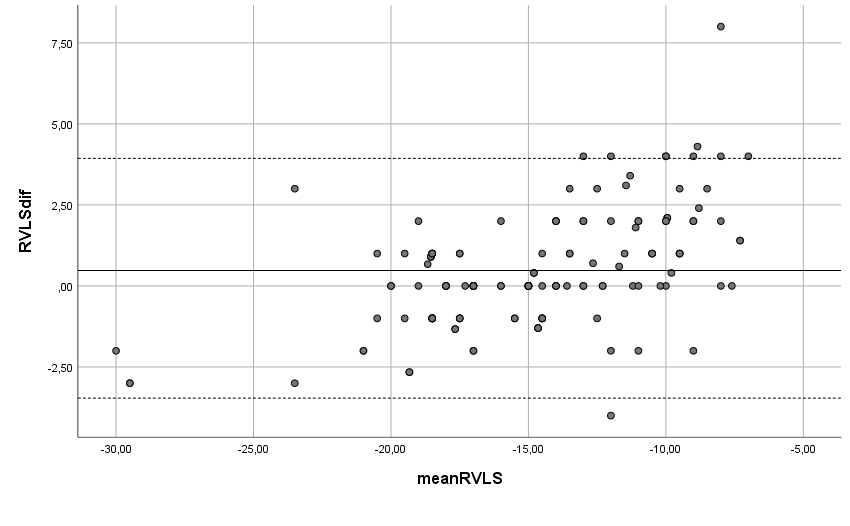


Bland & Altman scatter plot for RV-LS. Solid black line refers to the mean difference (0.4758), dashed line illustrates the upper and lower bound of the 95% confidence interval of the difference.


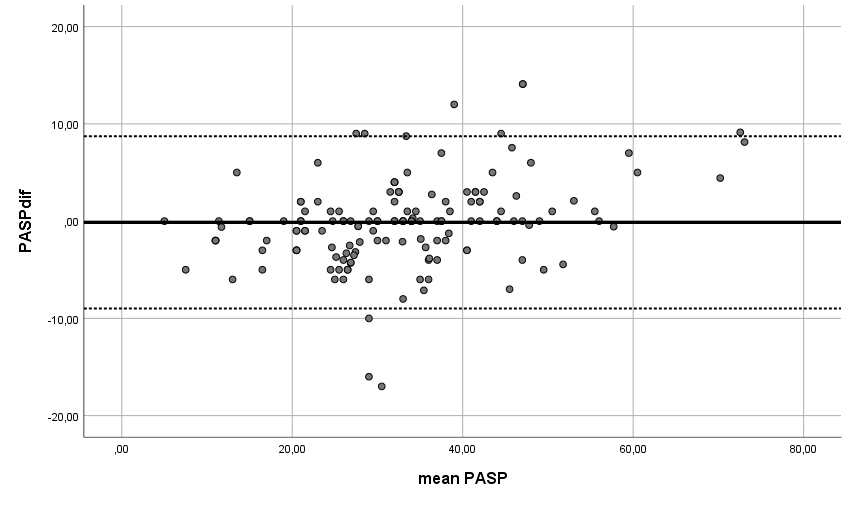


Bland & Altman scatter plot for PASP. Solid black line refers to the mean difference (-0.1313), dashed line illustrates the upper and lower bound of the 95% confidence interval of the difference.


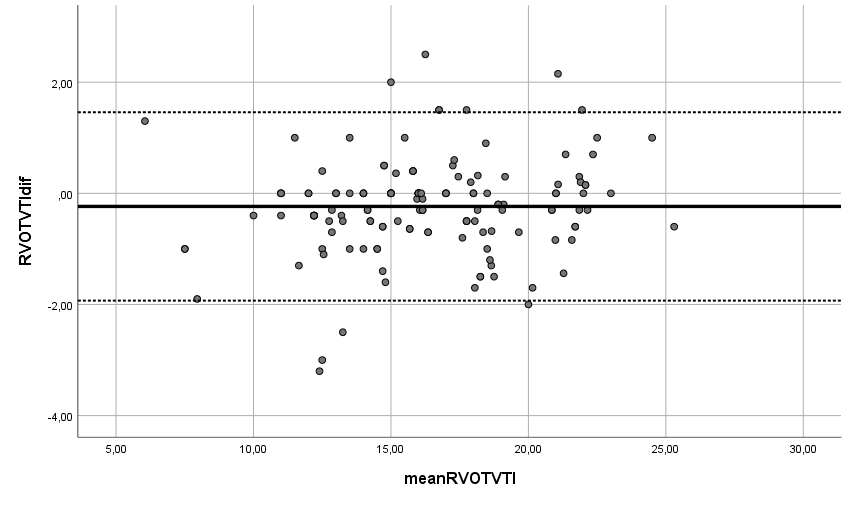


Bland & Altman scatter plot for RVOT VTI. Solid black line refers to the mean difference (0,2358), dashed line illustrates the upper and lower bound of the 95% confidence interval of the difference.


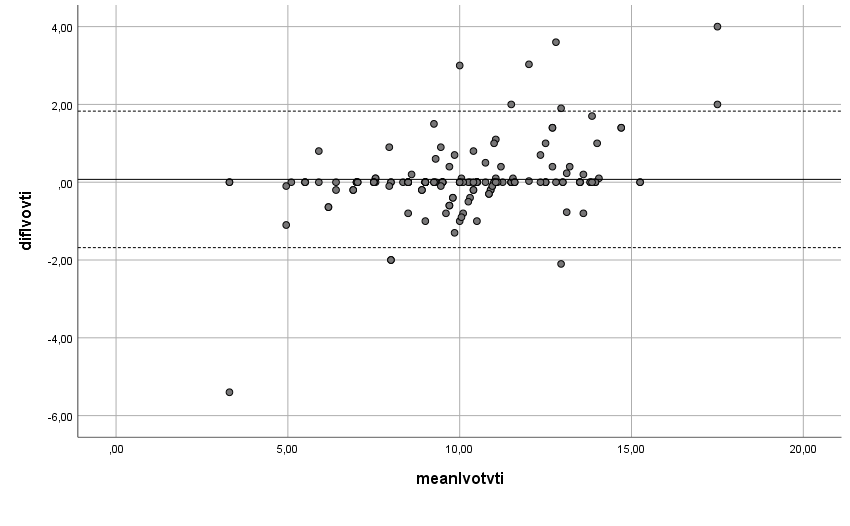


Bland & Altman scatter plot for LVOT VTI. Solid black line refers to the mean difference (0,0730), dashed line illustrates the upper and lower bound of the 95% confidence interval of the difference.


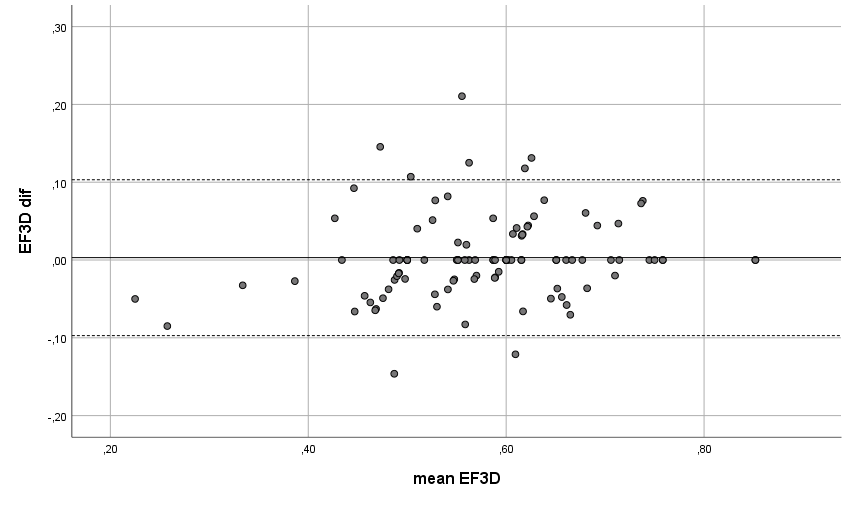


Bland & Altman scatter plot for EF (3D). Solid black line refers to the mean difference 0,003), dashed line illustrates the upper and lower bound of the 95% confidence interval of the difference.


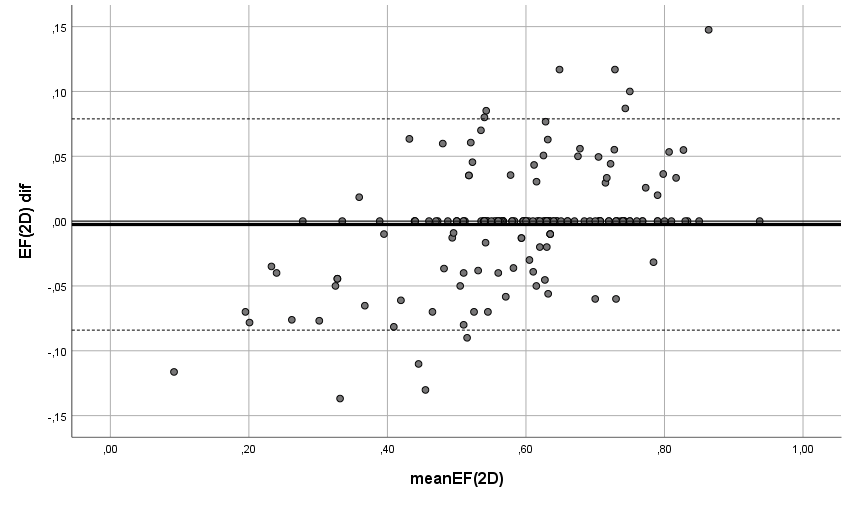


Bland & Altman scatter plot for EF (2D). Solid black line refers to the mean difference (-0,0026), dashed line illustrates the upper and lower bound of the 95% confidence interval of the difference.

**Table S8.**

**Clinical Characteristics and Echocardiographic Variables in patients stratified according to the troponin value upon admission. Echocardiographic data upon re-evaluation on the 10^th^ ICU day.**

|  | **TNI≤0.04 ng/ml (n=90)** | | | **TNI > 0.04 ng/ml (n=86)** | | |
| --- | --- | --- | --- | --- | --- | --- |
|  | **Admission** | **Day 10** | **p-value** | **Admission** | **Day 10** | **P-value** |
| **Echocardiographic variables: left ventricle** | | | | | | |
| LVEDD, cm | 4.64 ± 0.08 (71) | 4.58 ± 0.08 (71) | 0.765 | 4.53 ± 0.08 (56) | 4.55 ± 0.06 (56) | 0.897 |
| IVS, cm | 0.9 ± 0.01 (71) |  |  | 0.92 ± 0.01 (56) |  |  |
|  |  |  |  |  |  |  |
| LVEDA, cm^2^ | 29.5± 0.7 (71)* | 28.4± 0.8 (71) | 0.413 | 26.9± 0.6 (56) | 26.5± 0.7 (56) | 0.162 |
| LVESA, cm^2^ | 17.7± 1.1 (71) | 14.8± 0.9 (71) | 0.413 | 14.9 ± 1.1 (56) | 13.1± 0.8 (56) | 0.162 |
| VTI*_LVOT_*, cm | 20± 0.6 (71) | 20.7± 0.5 (71) | 0.413 | 20.4± 0.5 (56) | 20.9± 0.5 (56) | 0.162 |
| LVEDV, ml (2D) | 90.3 ± 3.5 (71)* | 85.1 ± 3.3 (71) | 0.398 | 82.7± 3.3 (56) | 81.5 ± 3.1 (56) | 0.327 |
| LVESV, ml (2D) | 38.8 ± 3.1 71) | 35.4 ± 2.1 (71) | 0.777 | 35.3 ± 2.5 (56) | 29.5 ± 1.9 (56) | 0.836 |
| EF (2D), % | 59.7 ± 1.7 (71) | 58.1± 2.1 (71) | 0.253 | 58.2 ± 1.6 (56) | 63.4 ± 2.1 (56) | 0.341 |
| SV, ml (Simpson’s) | 49.5± 2.4 (71) | 49.7± 2.3 (71) | 0.825 | 45.8± 2.8 (56) | 51± 2.5 (56) | 0.273 |
| LVEDV, ml (3D) | 86.9± 3.4 (38) | 88.1± 2.6 (38) | 0.763 | 78.7± 3 (34) | 80.9 ± 3.5 (34) | 0.097 |
| LVESV, ml (3D) | 37.5 ± 2.5 (38) | 36.7 ± 2.1 (38) | 0.822 | 31.3 ± 1.9 (34) | 34.1 ± 1.8 (34) | 0.152 |
| SV, ml (3D) | 49.1± 2.2 (38) | 51.5± 2.1 (38) | 0.658 | 45.7± 2.1 (34) | 48.2 ± 2.5 (34) | 0.464 |
| EF (3D), % | 57±1.7 (38) | 58.2±1.8 (38) | 0.765 | 58.3±1.6 (34) | 59.1±1.8 (34) | 0.524 |
| LV-LS, % | -13.2 ± 0.5 (57) | -14.1 ± 0.7 (57) | 0.101 | -13.4 ± 0.5 (42) | -14.4 ± 0.6 (42) | 0.068 |
| LV S’, cm/s | 10.1 ± 0.7 (54) | 10.3 ± 0.7 (54) | 0.269 | 10.5 ± 0.7 (42) | 10.7 ± 0.7 (42) | 0.103 |
| **Right Ventricle** | | | | | | |
| RVEDA/LVEDA | 0.78± 0.03 (71)* | 0.79± 0.03 (71) | 0.3 | 0.84± 0.03 (56) | 0.82± 0.05 (56) | 0.554 |
| RVEDA, cm^2^ | 22.4 ± 0.7 (71) | 21.9 ± 0.9 (7) | 0.3 | 22.03 ± 0.6 (56) | 20.7 ± 1 (56) | 0.126 |
| RVESA, cm^2^ | 13.9 ± 0.4 (71) | 13.3 ± 0.7 (71) | 0.103 | 13.9 ± 0.4 (56) | 12.9 ± 0.8 (56) | 0.110 |
| RV FAC, % | 36.6 ± 1.4 (71) | 40.6 ± 1.6 (71) | 0.05 | 36.2 ± 1.4 (56) | 39.2 ± 1.9 (56) | 0.167 |
| RVEDV*^b^*, ml | 107.9 ± 7.7 (37) | 107.1 ± 6.9 (37) | 0.420 | 106.2 ± 4 (36) | 118.9 ± 6.7 (36) | 0.983 |
| RVESV*^b^*, ml | 68 ± 6.1 (37) | 64.8 ± 4.3 (37) | 0.323 | 61.4 ± 2.8 (36) | 68.3 ± 4.3 (36) | 0.320 |
| RVEF*^b^* (%) | 39.2 ± 2.2 (37) | 39.5 ± 1.9 (37) | 0.624 | 42.4 ± 1.7 (36) | 42.6 ± 1.7 (36) | 0.097 |
| RV SV*^b^*, ml | 41.4± 3.3 (37) | 42.2 ± 3.2 (37) | 0.895 | 44.6 ± 2.2 (36) | 50.6 ± 3.5 (36) | 0.291 |
| PASP, mmHg | 33.3 ± 1.6 (66) | 28.9 ± 1.6 (66) | 0.011 | 35.8 ± 2.4 (43) | 30.3 ± 2.1 (43) | 0.033 |
| PASP/VTI*_LVOT_*, mmHg/cm | 1.9 ± 0.1 (66) | 1.5 ± 0.1 (66) | 0.021 | 1.8 ± 0.2 (42) | 1.6 ± 0.1 (42) | 0.015 |
| VTI*_RVOT,_* cm | 18.1 ± 0.5 (61)* | 17.5 ± 0.5 (61) | 0.595 | 16.6 ± 0.4 (40) | 17.4 ± 0.5 (40) | 0.021 |
| Pulmonary AcT, msec | 67.2 ± 2.1 (61) | 73.8 ± 2.7 (61) | 0.051 | 63.2 ± 2 (40) | 68.7 ± 3.3 (40) | 0.073 |
| RVOT notch | 17/61 (28.9%) | 8/61 (13.1%) | 0.159 | 19/40 (47.5%) | 9/40 (22.5%) | 0.096 |
| VAC_R,_ mm/mmHg | 0.76 ± 0.08 (61) | 0.97 ± 0.1 (61) | 0.024 | 0.84 ± 0.2 (40) | 0.98± 0.2 (40) | 0.294 |
| TAPSE, mm | 20±0.5 (68) | 22.4±0.2 (68) | 0.263 | 20.9±0.6 (56) | 21.1±0.5 (56) | 0.307 |
| RV S’, cm/sec | 15.6 ± 0.6 (71) | 16.4 ± 0.6 (71) | 0.574 | 16.6 ± 0.6 (56) | 16.4 ± 0.7 (56) | 0.655 |
| RV-LS, % | -14.3 ± 0.6 (61) | -14.8 ± 0.7 (61) | 0.407 | -14.7 ± 0.5 (45) | -14.2 ± 0.7 (45) | 0.819 |
| **Pericardial effusion** | 23 (25.6%)* |  |  | 47 (54.7%) |  | <0.0001 |
| *Maximum diameter measured during inspiration under Mechanical Ventilation.  Data are expressed as mean ± standard error of means; *^a^*  numbers in parenthesis refer to the actual number of patients that the variable was measured, ^b^ 3D measurements.  EF: Ejection Fraction; ΔIVC: respiratory variability in Inferior Vena Cava diameter [(IVCmax-IVCmin)/IVCmin]; LV-LS, longitudinal strain of the left ventricle; IVC, Inferior Vena Cava; IVS, Interventricular septum; LVEDD, Left Ventricular End Diastolic Diameter; LVEDV, Left Ventricular End Diastolic Volume; LVESV, Left Ventricular End Systolic Volume; LV s’, Systolic tissue doppler velocity measured at the lateral mitral annulus; PASP, Pulmonary Artery Systolic Pressure; PASP/VTI*_LVOT_*, Pulmonary Artery Systolic Pressure to Left Ventricular Outflow Tract Velocity Time Integral ratio; PASP/VTI*_RVOT_*, Pulmonary Artery Systolic Pressure to Right Ventricular Outflow Tract Velocity Time Integral ratio; RVEDA/LVEDA, Right Ventricular End Diastolic Area to Left Ventricular End Diastolic Area; RVEDV, Right Ventricular End Diastolic Volume; RVEF, Right Ventricular Ejection Fraction; RVESV, Right Ventricular End Systolic Volume; RVFAC, Right Ventricular Fractional Area Change; RV-LS, right ventricular free wall longitudinal strain; RV S’, Systolic tissue doppler velocity measured at the lateral tricuspid annulus; RV SV, Right Ventricular Stroke Volume; SV: Stroke Volume; TAPSE: Tricuspid Annular Plane Systolic Excursion; VAC*_R_*, Right Ventricular to Pulmonary artery coupling; VTI*_LVOT_*, Left Ventricular Outflow Tract Velocity Time Integral; VTI*_RVOT,_*  Right Ventricular Outflow Tract Velocity Time Integral; | | | | | | |
